# Trapping of the transport-segment DNA by the ATPase domains of a type II topoisomerase

**DOI:** 10.1038/s41467-018-05005-x

**Published:** 2018-07-03

**Authors:** Ivan Laponogov, Xiao-Su Pan, Dennis A. Veselkov, Galyna B. Skamrova, Trishant R. Umrekar, L. Mark Fisher, Mark R. Sanderson

**Affiliations:** 1 Randall Centre for Cell and Molecular Biophysics, 3rd Floor New Hunt’s House, Faculty of Life Sciences and Medicine, King’s College London, London, SE1 1UL UK; 2 Molecular and Clinical Sciences Research Institute, St. George’s, University of London, Cranmer Terrace, London, SW17 0RE UK; 3 Present Address: Department of Surgery and Cancer, Faculty of Medicine, Imperial College London, Sir Alexander Fleming Building, London, SW7 2AZ UK; 4 Present Address: The Institute of Structural and Molecular Biology, Department of Biological Sciences, Birkbeck College, University of London, Malet St., London, WC1E 7HX UK

## Abstract

Type II topoisomerases alter DNA topology to control DNA supercoiling and chromosome segregation and are targets of clinically important anti-infective and anticancer therapeutics. They act as ATP-operated clamps to trap a DNA helix and transport it through a transient break in a second DNA. Here, we present the first X-ray crystal structure solved at 2.83 Å of a closed clamp complete with trapped T-segment DNA obtained by co-crystallizing the ATPase domain of *S. pneumoniae* topoisomerase IV with a nonhydrolyzable ATP analogue and 14-mer duplex DNA. The ATPase dimer forms a 22 Å protein hole occupied by the kinked DNA bound asymmetrically through positively charged residues lining the hole, and whose mutagenesis impacts the DNA decatenation, DNA relaxation and DNA-dependent ATPase activities of topo IV. These results and a side-bound DNA-ParE structure help explain how the T-segment DNA is captured and transported by a type II topoisomerase, and reveal a new enzyme–DNA interface for drug discovery.

## Abstract (web summary)

Type 2 topoisomerases alter DNA supercoiling by an ATP-dependent reaction involving DNA passage through a cleaved DNA duplex. Here the authors present the crystal structure of the ParE ATPase domains of *S. pneumoniae* topoisomerase IV, in complex with a captured DNA oligonucleotide.

## Introduction

**Fig. 1:**
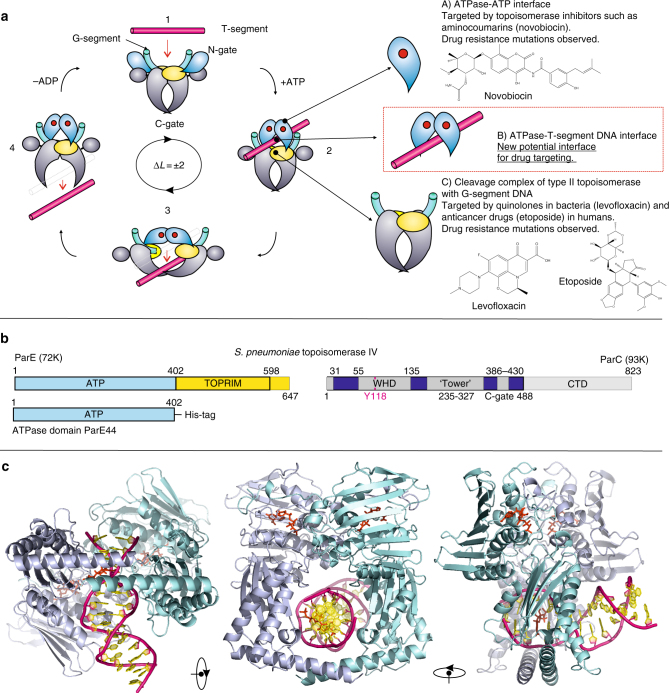
T-segment DNA capture and transport by type IIA topoisomerases. **a** The proposed type II topoisomerase reaction cycle exemplified by topoisomerase IV. ParC subunits are in grey, ParE N-terminal domain is in cyan, ParE C-terminal TOPRIM domain (containing the metal binding TOPRIM fold) is in yellow. G-gate DNA is in green and the transported T-segment DNA is in mauve. ATP bound to the ATPase domain is denoted by a red dot. Drug-targetable domains within the type II topoisomerase complex are highlighted in subsections A, B and C with examples on the right-hand side of the figure. **b** Domain organization of *S. pneumoniae* topoisomerase IV. The active enzyme is a ParC_2_ParE_2_ tetramer. The ATPase domain (residues 1–402) is shown in light blue. **c** Orthogonal views of the ParE44 protein in complex with a captured 14-mer T-segment DNA. N-terminal ParE domains are shown in cartoon mode in light blue and cyan, bound AMP-PNP molecules are in red and T-segment DNA is in mauve/yellow for backbone/bases, respectively

Changes to the topological state of DNA are required for many DNA transactions including DNA replication and chromosome segregation^1–3^. Topoisomerases are ubiquitous enzymes that act to alter DNA topology and are the targets of clinically important antibacterial and anticancer drugs^1–7^. Bacteria usually express two highly conserved type IIA topoisomerases (Top2As), topoisomerase (topo) IV and DNA gyrase, involved respectively in chromosome relaxation/segregation and DNA supercoiling (Fig. 1)^8–11^. Topo IV is a tetramer composed of two subunits, ParC and ParE, that form an active E_2_C_2_ complex (Fig. 1); likewise gyrase through GyrA and GyrB subunits^12^. Topo2As introduce a transient 4 bp staggered break into one DNA duplex and pass a second DNA duplex through the break which is then resealed (Fig. 1)^13,14^. The transported DNA (termed the T segment) is thought to be captured by closure of the N gate (ATPase gate) involving adenosine triphosphate (ATP)-dependent dimerization of the ParE/GyrB ATPase domains allowing T-segment presentation to the cleavage core of the enzyme, passage through the G (gate) segment DNA bound at the DNA cleavage gate and subsequent release through the protein C gate (Fig. 1a)^3–7,15,16^. Antibacterial coumarins such as novobiocin target the ATPase gate, whereas clinically important fluoroquinolones such as levofloxacin interfere with DNA resealing at the DNA cleavage gate (Fig. 1a)^17–22^.

The ParE and GyrB proteins exhibit the same modular design with an N-terminal ATPase domain and a C-terminal magnesium-binding (TOPRIM) domain (Fig. 1b)^3,12,23^. Similarly, the ParC and GyrA proteins are constructed of an N-terminal breakage-reunion domain with winged helix fold, tower and C gate, and a DNA-binding C-terminal domain (CTD)^3,12^. Interestingly, eukaryotic Top2As are homodimers in which all four domains are present in each subunit organized in a ParE–ParC arrangement^12^. In mammalian cells, the enzyme is expressed as distinct topo IIα and topo IIβ isoforms whose DNA cleavage gates are inhibited by clinically important anticancer drugs such as doxorubicin and etoposide (Fig. 1a)^24,25^. It appears that prokaryotic and eukaryotic Top2As share a similar double-strand break mechanism utilizing the sequential operation of N (ATPase), DNA and protein C gates (Fig. 1a)^3–5^. Structural and biochemical experiments employing topo II inhibitors have been particularly informative about the DNA cleavage gate. However, the nature of the T‑segment DNA and how it is captured for transport by type II topoisomerases have been longstanding questions in the field.

In this study, we have co-crystallized the ParE ATPase domain with short DNA duplexes in the presence of adenosine 5'-[β,γ-imido]triphosphate Adenylyl-imidodiphosphate (AMP-PNP), a non-hydrolysable ATP analogue that induces dimerization of ATPase domains to close the ATPase gate. We report the first co-crystal structure of a topoisomerase ATPase complex with T-segment DNA captured inside a cavity formed by the protein dimer. The structure represents the first visualization of this key intermediate in the Top2A reaction pathway. In concert with mutagenesis and biochemical analysis of strand passage and ATPase stimulation (plus a second ParE structure involving side-bound DNA), our work provides new perspectives on the mechanism of DNA capture and passage by type IIA topoisomerases.

## Results

### Structures of ParE-DNA complexes

To capture a closed ATPase clamp with prospective T segment, we screened a variety of DNA duplexes in co-crystallization trials with the *Streptococcus pneumoniae* ParE44 ATPase domain (residues 1–402) (Fig. 1b). Crystals were obtained with ParE44 in the presence of AMP-PNP using either a 14-mer DNA duplex, 5′-GCATATATATATGC-3′ or the 6-mer DNA duplex, 5′-GCGCGC-3′, and both X-ray structures were solved by molecular replacement revealing two different DNA binding modes (Table 1).

**Table 1:** Data collection and refinement statistics

	T-segment DNA—N-terminal ParE44 through-hole DNA bound complex (5J5Q)	T-segment DNA—N-terminal ParE44 side-bound DNA complex (5J5P)
*Data collection*		
Space group	*C*2	*C*222_1_
*Cell dimensions*		
*a*, *b*, *c* (Å)	124.95, 72.74, 222.96	134.44, 136.46, 136.27
*α*, *β*, *γ* (°)	90.00, 91.89, 90.00	90.00, 90.00, 90.00
Resolution (Å)	60.76–2.83 (2.92–2.83)^a^	45.18–1.97 (2.04–1.97)^a^
*R*_sym_ or *R*_merge_	0.047 (0.466)	0.063 (0.367)
Rpim	0.054 (0.448)	0.049 (0.225)
CC1/2	0.993 (0.85)	0.999 (0.952)
*I*/*σI*	5.3 (1.5)	6.4 (1.7)
Completeness (%)	99.7 (99.8)	99.8 (99.8)
Redundancy	3.3 (3.3)	6.7 (7.0)
AU composition	Two protein biological dimers of ParE44 are present per AU. Only one of them contains interpretable electron density corresponding to the bound DNA duplex (through-hole binding).	One biological dimer of ParE44 is present per AU. Each biological dimer of ParE44 is bound by two DNA duplexes (side binding).
*Refinement*		
Resolution (Å)	60.76–2.83	45.18–1.97
No. reflections	48,001	88,222
*R*_work_/*R*_free_	18.37%/26.30%	15.05%/17.40%
No. atoms		
Protein	11,436	6045
DNA	568	480
Ligand/ion	151	94
Water	–	824
B factors		
Protein	77.62	27.34
DNA	224.79	68.63
Ligand/ion	84.32	19.41
Water	–	40.47
R.m.s. deviations		
Bond lengths (Å)	0.026	0.017
Bond angles (°)	2.330	1.211

^a^Values in parentheses are for highest-resolution shell

The structure of the ParE44–14-mer DNA complex (PDB 5J5Q) was solved in space group C2 at 2.83 Å (Fig. 1c, Table 1). Two ParE44 dimers were present in the asymmetric unit (AU): one dimer had a trapped T-segment DNA duplex in its central cylindrical hole (Fig. 1c) (with evidence of weaker hole-bound density in the second dimer, likely washed out by the two possible alternative binding modes of the palindromic DNA to the second protein dimer). The structure shows unequivocally that DNA can be captured in the closed ATPase gate per se, a key mechanistic issue left open by earlier topoisomerase ATPase structures, all of which lack DNA^26–32^.

ParE44 folds into two subdomains (Fig. 1c) as seen for the related *Escherichia coli* GyrB43:^26^ an N-terminal proximal domain (residues 1–227, commonly referred to as the GHKL ATPase domain) formed of an eight-stranded β-sheet supported by four α-helices, and a distal C‑terminal domain (residues 227–402, termed the transducer/RNAse P-like domain) comprising a four-stranded β-sheet supported by three α-helices in its core and further extended by a long C-terminal α-helix which forms the bottom gate-closing arms in the dimer, as seen for *E. coli* GyrB^26^. Both GHKL ATPase domains of the dimer have a bound AMP-PNP molecule and a coordinated magnesium ion. Residues 1–21 of each ParE44 monomer protrude towards ATP binding cavities on the opposite monomer within the dimer and sit like a lid above the adenine ring with the hydroxyls of Y-11 coming into close proximity with N3 of the adenine ring. On the other side, N3 is proximal to M-83 and Y-113 of the same side monomer. K-107 forms a salt-bridge with the β-phosphate of the AMP-PNP and the octahedral coordination of the nucleotide-bound Mg^2+^ is satisfied by oxygens of the three phosphates and the oxygen of residue N-51.

**Fig. 2:**
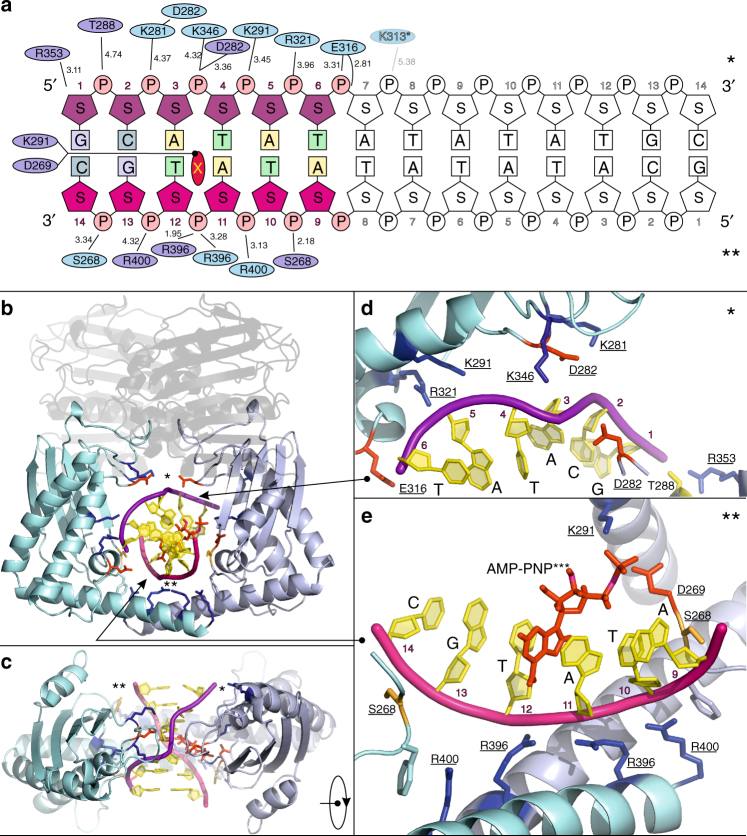
Molecular features of the ParE44 complex with hole-bound 14-mer DNA. **a** Amino acid side chains that interact with the DNA duplex are shown as ellipses with colouring corresponding to the two subunits of the ParE dimer. Shortest distances between the DNA and the amino acid side chains are given in Ångstroms. The 6 bp DNA section that interacts with ParE is coloured, the solvent-exposed region is not. The top and bottom DNA strands are in purple and pink and are marked by single and double asterisks. X denotes a putative DNA intercalating AMP-PNP molecule shown throughout in red. The potential weakly interacting amino acid K313 is greyed. **b**, **c** Front and top views of the DNA–protein interaction, with the uninvolved N-proximal GHKL ParE region greyed out. Arrows from/to the zoom-in **d**, **e** showing the top (*) and bottom (**) DNA strand interaction sites indicate the direction of views displayed in **d**, **e**. Residues targeted for mutagenesis are underscored

The 14-mer DNA duplex penetrates through the 22 Å hole in the protein formed by the ParE44 RNAse P-like domains (Figs. 1c and 2) and is mainly in the B form apart from a kinked region between basepairs 3 and 4 (i.e., between the first two AT and TA basepairs after the 5’-terminal GC and CG pairs). (Junctions between G-C and A-T bps are known to be highly flexible, one of the reasons for using the 14-mer sequence in crystallization.) The DNA experiences in total a complex and asymmetric 39.4° change of direction (see Supplementary Fig. 1 for details). The major and minor grooves of the duplex are oriented towards opposite RNAse P-like domains of the ParE44 dimer and are almost equalized in width due to kinking. The protein-interacting 6 bp segment of the 14-mer duplex DNA, 5′-GCATAT (Fig. 2a) is asymmetrically bounded by the RNAse P-like domains comprising an upper arch formed by protein residues in the region 269–291 and by the short helices comprising residues 316–322 at the end of the anti-parallel β-sheet which also in turn binds in the major and minor grooves of the double helix from both sides of the dimer (Fig. 2c). The two paired long C-terminal α-helices (residues 372–402) at the base of the dimer form the other side of the hole (Fig. 1c, Fig. 2b, c).

**Fig. 3:**
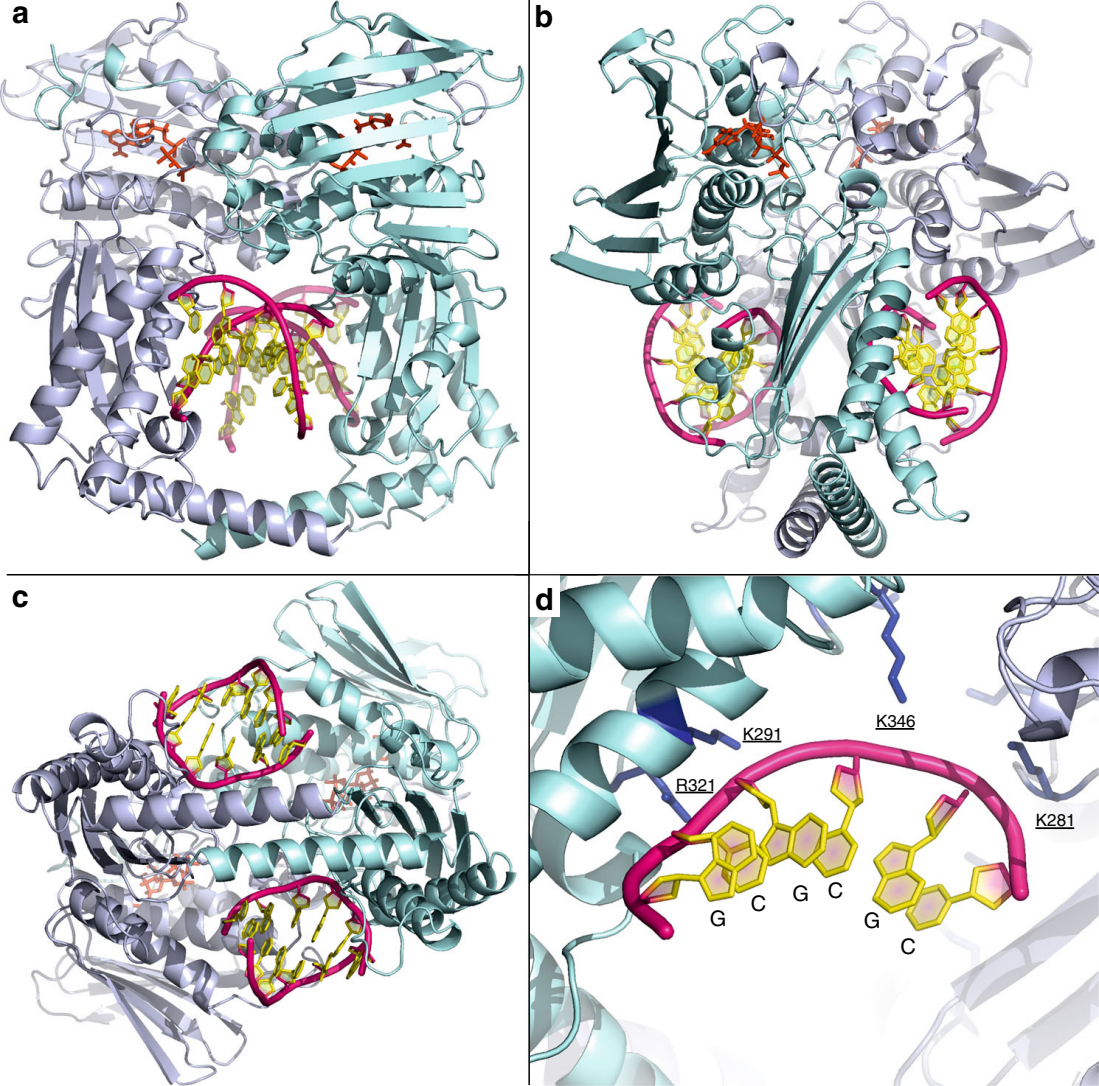
X-ray crystal structure of ParE44 with side-bound 5′-GCGCGC-3′ duplex DNA and AMP-PNP. **a**, **b**, **c** Front, side and bottom views of the complex and **d** residues that interact with the DNA and were targeted for mutagenesis (underscored). The ParE44 protein forms a closed dimer with two molecules of DNA bound as a regular B-form helix but on the outside of the protein rather than in the hole. N-terminal ParE domains are shown in cartoon mode in light blue and cyan, bound AMP-PNP molecules are in red and DNA is in mauve/yellow for backbone/bases, respectively

**Fig. 4:**
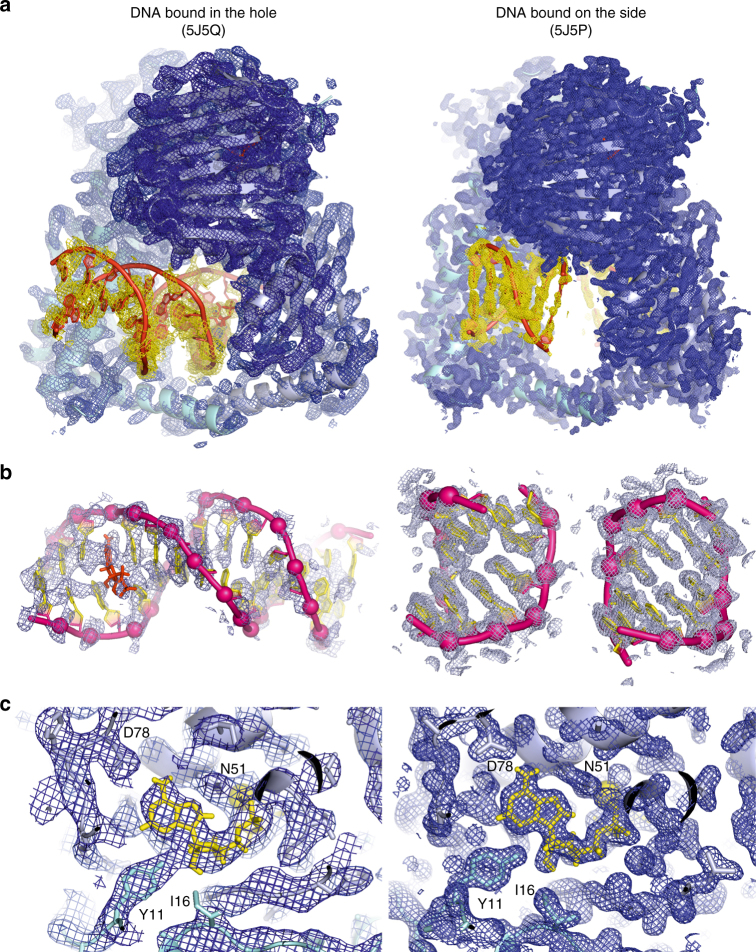
Comparison of hole-bound and side-bound ParE44-DNA structures. Composite omit electron density maps (2*F*_obs_−*F*_calc_) were contoured around **a** the entire molecular structure, **b** the bound DNA and **c** the bound AMP-PNP in the ATP binding site. Structures on the left-hand side represent the complex with the DNA bound in the hole formed by the ParE44 dimer. Structures on the right-hand side represent the side-bound DNA complex. In **a** the electron density map is contoured at 1.5 and 1.0 *σ* levels for protein and DNA in blue and yellow colours, respectively. In **b** the electron density map is contoured at 1.0 *σ* level and is in light blue. In **c** the electron density map is contoured at the 1.5 *σ* level only and is in blue. In **a** the protein and DNA are displayed in cartoon mode in light blue/cyan and red, respectively. In **b** the DNA backbone is in pink with phosphates indicated by spheres, the DNA bases are in yellow and the intercalated molecule X is in red. In **c** the bound AMP-PNP molecule and the coordinated magnesium are shown in yellow, while the protein residues are in light blue/cyan. Magnesium is shown as a sphere, side chains and AMP-PNP are shown using stick representation and the protein backbone is displayed in cartoon mode

Kinking of the DNA is a feature of the hole-bound DNA complex but is not seen in a second X-ray crystal structure for ParE44 co-crystallized with AMP-PNP but with a 5′-GCGCGC-3′ duplex solved at 1.97 Å (5J5P) (Fig. 3, Table 1). This second complex revealed two DNA helices bound outside of the hole one to each side of the ParE44 dimer. Both 6-mer helices are B form and unkinked. Comparison of the ParE hole in the 14-mer hole-bound and 6-mer outside-bound structures showed that hole binding by DNA did not significantly alter the dimensions of the cavity (Supplementary Fig. 2) and that the side-bound DNAs interact weakly with adjacent dimers in the crystal lattice (Supplementary Fig. 3). Surprisingly, we detected extra electron density located at the kink between basepairs 3 and 4 in the 14-mer DNA helix (designated by “X” in Fig. 2a) but not in the unkinked side-bound DNA structure (see Fig. 4 for the omit electron density maps contoured for the full complexes, for DNA and for AMP-PNP at its binding site). The extra density seen in the kinked 14-mer DNA (Fig. 4b and Supplementary Fig. 4) is consistent with the presence of an aromatic molecule. Reviewing the chemical composition of our crystallization and crystal freezing solutions, by exclusion, we tentatively ascribe the extra density to a molecule of AMP-PNP that is intercalated into the DNA (Fig. 2c, e), a feature that, to our knowledge, has not been seen previously. Confirmation will require a higher resolution structure.

Additional information on DNA kinking in the hole-bound ParE44-DNA structure arises from the 14-mer being palindromic and therefore carrying the 5′-GCATAT ParE-binding site at both ends of the molecule (Fig. 2a). The structure reveals that one DNA end is bound in the ParE hole and is bent; the other 5′-GCATAT DNA end is not bound to protein, protrudes into solvent, adopts a B-form helix and is unbent (Figs. 2 and 4, Supplementary Fig. 5). For the binding and bending to be crystallographic artefacts, the DNA would need to be involved in crystal contacts. Importantly, in the crystal lattice, the DNA forms its only detectable protein contacts with the hole of the protein dimer to which it is bound and not to crystallographically related molecules (Supplementary Fig. 5). These results provide strong evidence that the 5’-GCATAT sequence can adopt two conformations—a B-form helix in solution that becomes bent when bound inside the ParE hole.

### Cavity residues are involved in DNA strand passage activity

**Fig. 5:**
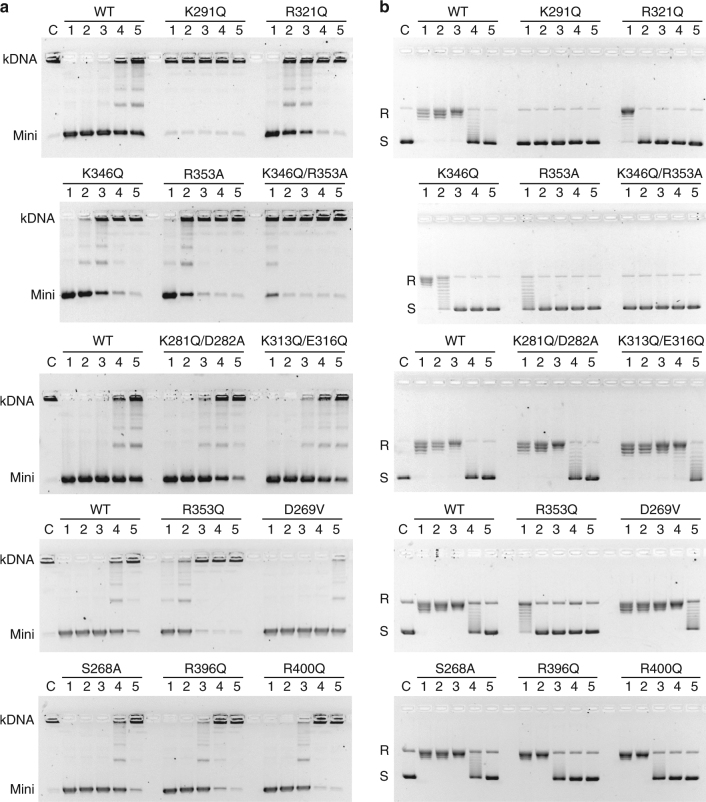
Mutations of ParE cavity residues affect DNA strand passage by topo IV. Complexes of topo IV reconstituted with ParE bearing cavity mutations at residues K291, R321, K346 or R353 exhibit greatly reduced DNA decatenation (**a**) and DNA relaxation (**b**) activities. **a** A representative decatenation experiment. For each panel, kinetoplast DNA was incubated at 37 °C for 1 h in decatenation buffer (40 mM Tris-HCl (pH 7.5), 6 mM MgCl_2_, 10 mM DTT, 200 mM potassium glutamate, 50 µg ml^−1^ BSA) with 1 mM ATP and *S. pneumoniae* topoisomerase IV reconstituted from a fixed amount of ParC (25 ng) (and in experiments not shown up to 2000 ng) and titrated with either wild-type or mutant ParE (as indicated) at 100, 20, 10, 5 and 2.5 ng (lanes 1–5, respectively in each panel). Reactions were terminated and the DNA products were separated and visualized by electrophoresis in 1% agarose gels. Lane C, no enzyme addition. kDNA, kinetoplast DNA; Mini denotes minicircle DNA released by topo IV. Intermediate bands are partially unlinked DNA species. **b** Relaxation assays for topo IV were carried as described for kDNA decatenation except supercoiled plasmid pBR322 DNA (400 ng) was used as substrate with the same level of ParC (25 ng) and wild-type or mutant ParE at 100, 40, 20, 10 and 5 ng (each panel lanes 1–5, respectively). DNA products were analysed on 1% agarose gels. Lane C, no enzyme addition. R and S, relaxed and supercoiled DNA, respectively

**Fig. 6:**
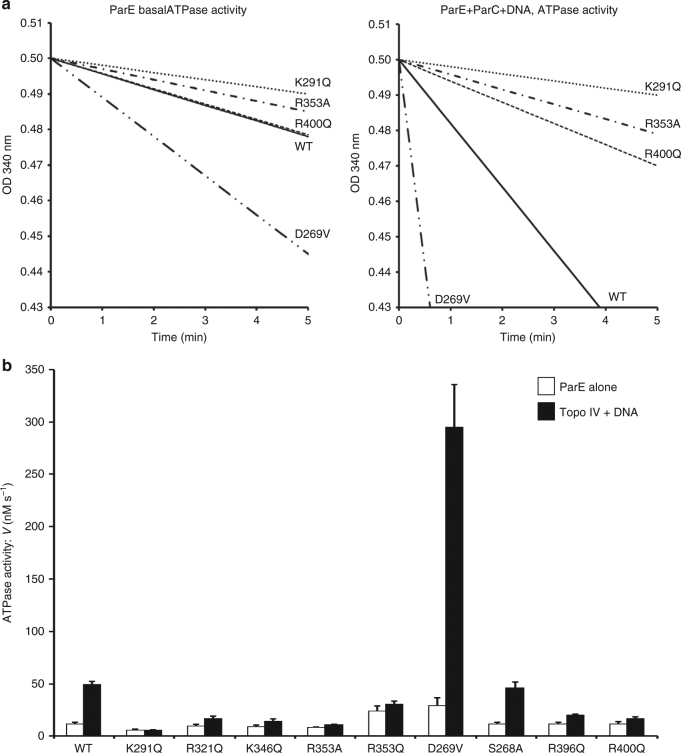
Measuring the ATPase activity of ParE proteins. **a** Comparison of basal ATPase rates for wild-type (WT) and representative ParE cavity mutants and stimulation by ParC and DNA. A coupled enzyme assay was used wherein ATPase activity was measured through the change in NADH absorbance at 340 nm^34,35^. The reaction mix contained topo IV decatenation buffer, 2 mM ATP and 100 nM ParE and was incubated at 37 °C in the presence or absence of 130 nM ParC and 10 µg pBR322 (final volume of 500 µl). **b** Bar chart showing the ATPase activity of WT ParE and various single mutants of ParE observed in the absence and presence of DNA and ParC (to form topo IV). Reactions were carried out in triplicate on separate days. Error bars show the standard error. Inclusion of DNA or ParC alone did not stimulate ParE ATPase activity. The ParE D269V mutant showed strong basal ATPase activity that was greatly enhanced by inclusion of ParC and DNA

**Fig. 7:**
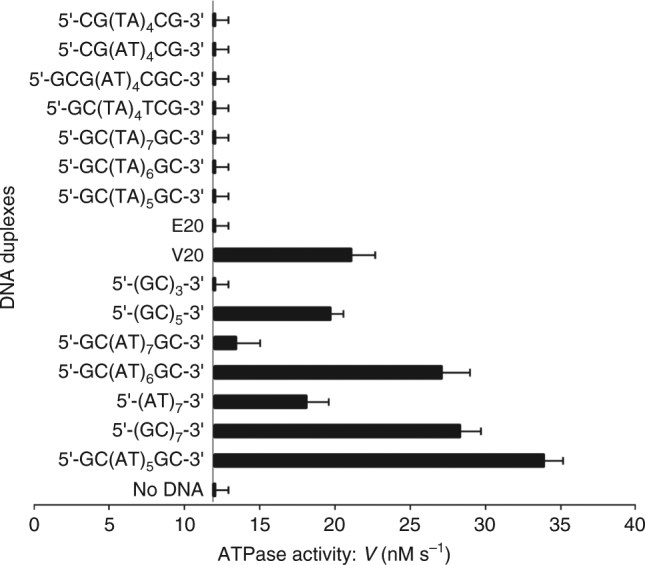
Short DNA duplexes stimulate ATP hydrolysis by topoisomerase IV. Using the conditions described in the legend to Fig. 6, topo IV was incubated with 2 mM ATP in the absence and presence of a variety of short annealed oligonucleotide duplexes (10 µg) and ATP hydrolysis was followed by a coupled assay. The results shown are the average of three independent measurements. In the absence of DNA, the basal ATPase activity of topo IV was 12.4 nM s^−1^. Asymmetric E20 and V20 are 20-mers of the E and V sites that bind as G (gate DNA) segments and are strongly cleaved by topo IV^22,30^ and were produced in duplex form by annealing complementary strands as carried out for 5′-GC(TA)_4_TCG-3′

The ParE hole is lined with positively charged arginine and lysine residues that potentially interact with DNA backbone phosphates to stabilize the bound DNA (detailed in Fig. 2a). Interestingly, the DNA is bound asymmetrically in the hole. Thus, the top strand (asterisked in Fig. 2a, d) is bound by residues derived uniquely from one or other RNAse P-like domain in the dimer (but not both), in contrast to the more symmetric engagement of the bottom DNA strand (double asterisk) by pairs of residues S268, R396 and R400 contributed by both subunits (Fig. 2a, e). To examine the functional aspects of these interactions, full-length ParE proteins altered by individual isosteric mutation of the 12 cavity residues (Lysine or Arginine to Glutamine; Serine to Alanine, Aspartate to Valine) were produced by site-directed mutagenesis (Supplementary Table 1), overexpressed in soluble form and purified to homogeneity. Reconstitution with full-length ParC subunit produced the mutant topo IV complexes allowing analysis of DNA strand passage (Fig. 5), DNA cleavage (Supplementary Fig. 6) and ATPase activities (Figs. 6 and 7, Table 2).

**Table 2:** Activities of wild-type and mutant topo IV enzymes

Protein	Decatenation	Relaxation	Cleavage	Basal ParE ATPase	Topo IV+DNA, fold stimulation
Wild-type ParE	100%	100%	100%	100%	4.15
^*^ParE K291Q	No activity^a^	No activity^a^	100%	57%	1.0
^*^ParE R321Q	20–25%	20%	100%	86%	1.6
^*^ParE K346Q	30%	~25%	100%	72%	1.6
^*^ParE R353A	10%	10%	100%	72%	1.37
^*^ParE R353Q	10%	10%	100%	200%	1.3
^*^ParE K346Q/R353A	1%	<0.5%	100%	93%	1.2
^*^ParE K281Q/D282A	100%	100%	100%	74%	3.67
^*^ParE K313Q/E316Q	100%	>100%^b^	100%	65%	8.5
ParE D269V	200%	200%	100%	270%	10
^**^ParE S268A	100%	100%	100%	100%	4.0
^**^ParE R396Q	50%	50%	100%	100%	1.7
^**^ParE R400Q	50%	50%	100%	100%	1.4

Topoisomerase IV was reconstituted from full-length ParC and wt or mutant full-length ParE and their activities were determined as the average of three independent measurements. Single asterisk denotes complexes mutated in ParE residues that interact asymmetrically with the top strand of the DNA duplex (Fig. 2): double asterisk identifies complexes mutated in residues that interact symmetrically with the bottom DNA strand in Fig. 2

^a^No activity detectable using up to 2 µg of ParE

^b^About twofold more active than wild type. The basal ATPase activity of wt and mutant ParE proteins was novobiocin sensitive (Supplementary Table 2)

Mutation of DNA binding residues K291, R321, K346 or R353 (lining the top arch of the ParE hole, Fig. 2a, d) abrogated or greatly reduced the ATP-dependent DNA decatenation activity of topo IV, whereas double changes in more distantly placed residues K281/D282 and K313/E316 were without effect (Fig. 5a, top three panels; Table 2). Interestingly, mutation of R396 and R400 residues (from the crossed C-terminal α-helices) had modest (twofold) inhibitory effects (Fig. 5a, bottom panel), with wild-type activity observed for the S268A mutant. In addition, a valine mutation of D269 (the residue that binds the DNA-intercalating molecule) conferred hyperactivity in decatenation (Fig. 5a, fourth panel, Table 2). Essentially the same pattern of results was seen for DNA relaxation, a second strand passage activity, except for the additional twofold stimulation of DNA relaxation seen for the double change of K313 and E316) (Fig. 5b, Table 2). An important control was that all 12 topo IV complexes mutated in the DNA binding ParE cavity retained full ATP-independent quinolone-promoted DNA cleavage activity (Supplementary Fig. 6), indicating that the mutant ParE proteins are properly folded and behave normally in activating ParC. Thus, these comprehensive mutagenesis experiments indicate that ParE cavity residues, particularly K291, R321, R346 and R353, are crucial to strand passage activity consistent with a role in capturing T-segment DNA for transfer through the DNA cleavage core.

### Cavity mutations affect topo IV ATPase activity

Top2As exhibit both intrinsic (basal) and DNA-activated ATPase activities^33^ but little is known about the protein and DNA sequence requirements. We found that the basal activity of ParE proteins was novobiocin sensitive (Supplementary Table 2) (showing it was intrinsic to ParE) and, as also found for GyrB^33–35^, was not enhanced by ParC or DNA alone, but required the addition of both for DNA-stimulated activity. To explore the relation between DNA binding and ATP hydrolysis, we examined the effect of 12 ParE cavity mutations on the basal ATPase activity of ParE and of topo IV using pBR322 DNA as substrate (representative results are shown in Fig. 6 and summarized for all the mutant proteins in Table 2). Wild-type topo IV exhibited a basal ATPase activity that was stimulated some fourfold by addition of linear or supercoiled plasmid DNA (Fig. 6, Table 2). K291Q, R321Q, K346Q and R353A mutations reduced both the basal ATPase activity of ParE and especially the DNA-stimulated activity in the presence of ParC. Interestingly, the K291Q ParE protein had 60% of the basal wild-type (wt) ATPase but addition of DNA and ParC gave no stimulation consistent with the idea that DNA binding inside the ParE cavity is required for DNA-activated ATP hydrolysis (Fig. 6, Table 2). The K291Q change completely abrogated DNA strand passage by topo IV (Fig. 5, Table 2). Similar results were seen in a more limited study describing a single mutation of the equivalent residue in GyrB^34^. Mutations of residues S268, R396 and R400 associated with the long cavity helices had no or little effect on basal ATPase, whereas hyperactivating mutations at D269 and K313/E316 both stimulated the basal ATPase and greatly enhanced DNA activation (Fig. 6, Table 2). The various effects of topo IV mutations on ATPase activities closely paralleled those seen for decatenation and relaxation (Table 2). Thus, mutations of DNA binding ParE residues that strongly inhibited DNA decatenation and relaxation also strongly reduced basal and/or DNA-stimulated ATPase activity. These results indicate that DNA binding, ATP hydrolysis and strand passage are closely coupled.

### Short DNA duplexes stimulate ATPase activity

Given that the 5′-GCATATATATATGC-3′ duplex binds the ParE cavity as a potential T segment (Fig. 1c), we examined whether this and other duplex sequences are sufficient to activate ATP hydrolysis by topo IV. As seen for GyrB and the GyrB43 ATPase region^33–36^, DNA alone did not measurably stimulate the very low basal activity of ParE44 or ParE alone. However, by complementing ParE with ParC, we showed for the first time that short DNA duplexes with alternating purine-pyrimidine sequences are able to stimulate the ATPase of topo IV (Fig. 7). Activity required duplex DNA: no stimulation was seen with single-stranded DNA. The cavity-binding 5′-GC(AT)_5_GC-3′ duplex had the greatest effect—a 2.8-fold ATPase stimulation (Fig. 7) which compares favourably with an ~4-fold stimulation by the same weight of pBR322 (Table 2). Other duplexes were tested that were sequence variations on the 14-mer. Substituting the 5′ end GC repeats with CG, and/or substituting internal 5′ AT repeats with TA, or either decreasing or increasing the number of AT repeats all failed to stimulate topo IV ATPase activity (Fig. 7). Polymeric 5′ (GC)_*n*_ sequences were inactive when *n* = 3 (corresponding to the side-bound hexamer (Fig. 3)), but were partially active when *n* = 5 or 7 as was 5′-(AT)_7_. The E-site DNA sequence that binds strongly as a G segment to the topo IV DNA cleavage core did not stimulate ATPase activity, although the V-site, a second strongly cleaved sequence, was partially effective in ATPase stimulation (Fig. 7). These results provide the first evidence for any type II topoisomerase that short DNA duplexes including the prospective T-segment 14-mer (Fig. 2a) are able to stimulate the ATPase activity, an observation with potentially important mechanistic implications.

## Discussion

Type IIA topoisomerases function as ATP-driven molecular machines that mediate the capture of a transported (T segment) DNA and its passage through a transient double-stranded DNA break in a second DNA duplex (the G segment)^1–7^. This process involves the sequential operation of a series of molecular gates, i.e., the N, G and C gates^1–7,15,16^. How the enzymes engage a T-segment DNA and how its capture by the N gate leads to strand passage has long been a key unresolved issue. Our structure of a T-segment DNA trapped in the cavity formed by dimerization of the nucleotide-bound ParE ATPase region of *S. pneumoniae* topo IV (Figs. 1 and 2) is the first structure to visualize this transient intermediate yielding new mechanistic insights on strand passage by type II topoisomerases. Interestingly, the ParE ATPase region introduces a kink into the T-segment DNA inducing a 40° bend, and moreover binds the DNA asymmetrically in the cavity through differential interactions with charged protein residues lining the hole (Figs. 1 and 2 and Supplementary Fig. 1). Mutation of the full set of residues that engage the T-segment DNA affected DNA strand passage and ATPase activities demonstrating that the crystallized complex is biologically relevant (Figs. 5 and 6, Table 2).

The ParE hole engages with a 6 bp sequence 5′-GCATAT-3′ found at both ends of the palindromic 14-mer DNA duplex (Fig. 2a). The sequence at one end is bound through the ParE hole and is bent, the other 5′ GCATAT end reaches out into the solvent and is an unbent B-form helix. Apart from interactions with the ParE dimer to which it is bound, there are no other detectable lattice interactions of the 14-mer with adjacent ParE dimers (Supplementary Fig. 5). This “internal control” suggests that kinking of the T-segment DNA occurs on binding to the ParE hole. This finding opens up the prospect of separate studies to detect T-segment bending by FRET or by gel retardation approaches.

One interesting aspect of the structure is that the 14-mer T-segment DNA duplex is readily accommodated by the 22 Å ParE cavity and by solvent channels in the crystal lattice (Supplementary Figs. 2 and 5). These features likely account for the higher DNA temperature factors (B factors) for the hole-bound DNA (224.79 Å^2^) compared to the protein (~78 Å^2^) and which increase along the DNA chain from ~190 Å^2^ for the hole-bound DNA end to ~260–350 Å^2^ for the solvent-exposed end (Table 1). These results suggest the DNA is less ordered than the protein consistent with loose DNA binding in the dimeric ParE annulus (Fig. 4). By comparison, for the side-bound DNA-ParE44 complex, the B factors on DNA and protein are 69 Å^2^ and 27 Å^2^, indicating tighter binding of the side-bound DNA (Table 1), possibly enhanced by DNA interactions between adjacent dimers in the crystal (Supplementary Fig. 3). Lack of tight DNA binding to a protein cavity is expected to be advantageous for enzymes such as type II topoisomerases where DNA movement or transport is central to catalysis. Indeed, the B factors are in line with other structures involving protein annulus–DNA interactions where the DNA is dynamic, for example, a proliferating cell nuclear antigen (PCNA)–DNA complex (PDB 3K4X) and SV40-T antigen complex (5CTC) wherein the B factors on the DNA are in the 300 Å^2^ range^37,38^, and the Ku protein–DNA complex (1JEY) where the B factors are in the 80–100 Å^2^ range^39^.

Mutagenesis of particular cavity residues identified from the 14-mer structure generally had parallel effects on the DNA decatenation, relaxation and ATPase activities of topo IV (Table 2). Thus, changes to residues K291, R321, K346 and R353 from the top arch of the cavity severely compromised both DNA strand passage and ATP hydrolysis confirming key roles of these residues in T-segment capture. Much weaker inhibitory effects (≤2-fold) were seen for mutagenesis of other residues such as S268 (no effect), and for R396 and R400 from the long alpha-helical arms forming the bottom of the cavity (Table 2). Hyperactivity in DNA strand passage and especially DNA-stimulated ATP hydrolysis was conferred for some ParE substitutions, notably by a valine substitution of D269, a residue that contacts the putative AMP-PNP intercalated at the DNA kink (Figs. 2 and 6, Supplementary Fig. 4, Table 2). Such hyperactive behaviour is not easily explained but could result from more efficient capture of the T segment due to the removal of the negatively charged protein residue from the vicinity of the putative phosphate position of the AMP-PNP.

Another new finding is that certain short DNA duplexes, including the hole-binding 14-mer, are sufficient to stimulate the topo IV ATPase activity whereas other duplexes, e.g., 5′-GCGCGC, were inactive (Fig. 7). ATPase stimulation likely arises from DNA binding to the T-segment storage cavity in the ParE ATPase. However, given that the full topo IV holoenzyme was used, it is also possible for some DNA duplexes that ATPase stimulation arose allosterically by DNA binding to the ParC CTD or to the DNA gate (although against this we note from Fig. 7 that the strongly cleaved E-site gate-DNA did not stimulate ATPase activity). Any binding to sites in ParC would complicate FRET analysis. As noted earlier, it is difficult to rationalise the outcomes in Fig. 7 using DNA sequence alone or on the basis of the ParE-14-mer structure which shows there are no direct protein contacts with DNA bases but rather interactions with the DNA backbone phosphates (Fig. 2a). Taken together, it is more likely that DNA conformation, e.g., the propensity for DNA bending rather than the DNA sequence per se, accounts for closely related DNA duplexes having different ATPase activating effects.

It was unexpected to recover a side-bound DNA-ParE complex in addition to that for the hole-bound bent DNA state (Figs. 1–4). Both complexes bind DNA asymmetrically through preferential interaction with one of the two subunits of the ParE44 dimer. The biological role of side-bound DNA is not immediately clear. However, importantly we note that the side binding of DNA to ParE occurs exclusively through K291, R321, and K346 and K281, i.e., an exact subset of the residues seen in the hole-bound DNA complex and shown by mutagenesis of 291, 321 and 346 to be important for enzyme activity (Figs. 2 and 3d, and Table 2). Lack of additional crystal lattice contacts for the bent DNA and weak crystal lattice interactions for the side-bound DNA (mainly allosteric contacts between major grooves of the crystallographically related DNA molecules) allows us to speculate that the two complexes represent consecutive stages of T-segment recognition and capture. We propose that binding first occurs through the side-bound DNA process and is crucial for initial stabilization of the T segment (reflected in the higher impact of mutation of the protein residues involved). Having bound to one of the ParE domains in the open clamp state, capture of the T segment occurs upon approach of the other domain and dimerization promoted by ATP. Engagement with other residues in the cavity, the possible high bendability of sequences such as 5′-GCATAT and putative intercalation would act to stabilize the T segment within the cavity.

The novel ParE-DNA structures (Figs. 1–3) give new perspective on several puzzling features of type II topoisomerase mechanism. First, asymmetric interaction of the captured T segment with the ParE subunits forming the ATPase hole (Fig. 2) provides an attractive mechanism for the observed stepwise hydrolysis of the two bound ATP molecules^40,41^. Secondly, the demonstrated ability of the ATPase hole to accommodate the T segment argues against an additional T-segment storage cavity proposed (on the basis of a low-resolution cryo-electron microscopy gyrase-DNA structure) to lie between the ATPase region and the cleavage core^42^. Third, in our closed clamp ParE44-DNA structures, the long C-terminal alpha-helices are crossed forming the bottom of the protein hole (Figs. 1–3). This feature is seen in a number of closed clamp topoisomerase structures but is absent in the open clamp state, raising questions as to whether and when twisting/untwisting of ATPase subunits takes place^30,31^. In the type II topoisomerase holoenzyme, it is perhaps unlikely that the helical arms would be crossed prior to T-segment passage as they would need to untwist again to allow T-segment escape and transport. Conceivably, the helical arms do not cross until after the T segment has passed, in which case the DNA contacts seen here with the crossed arms would be fortuitous. In support of this view, mutations in or close to the helical arms had weak inhibitory effects on topo IV DNA strand passage and DNA stimulated ATPase activities (Fig. 5, Table 2) consistent with minimal DNA contact with uncrossed helical arms and suggesting that ATPase subunit twisting with concomitant long arm crossing occurs after T-segment transit on to the cleavage core, as proposed in a recent model by Martinez-Garcia et al.^43^.

**Fig. 8:**
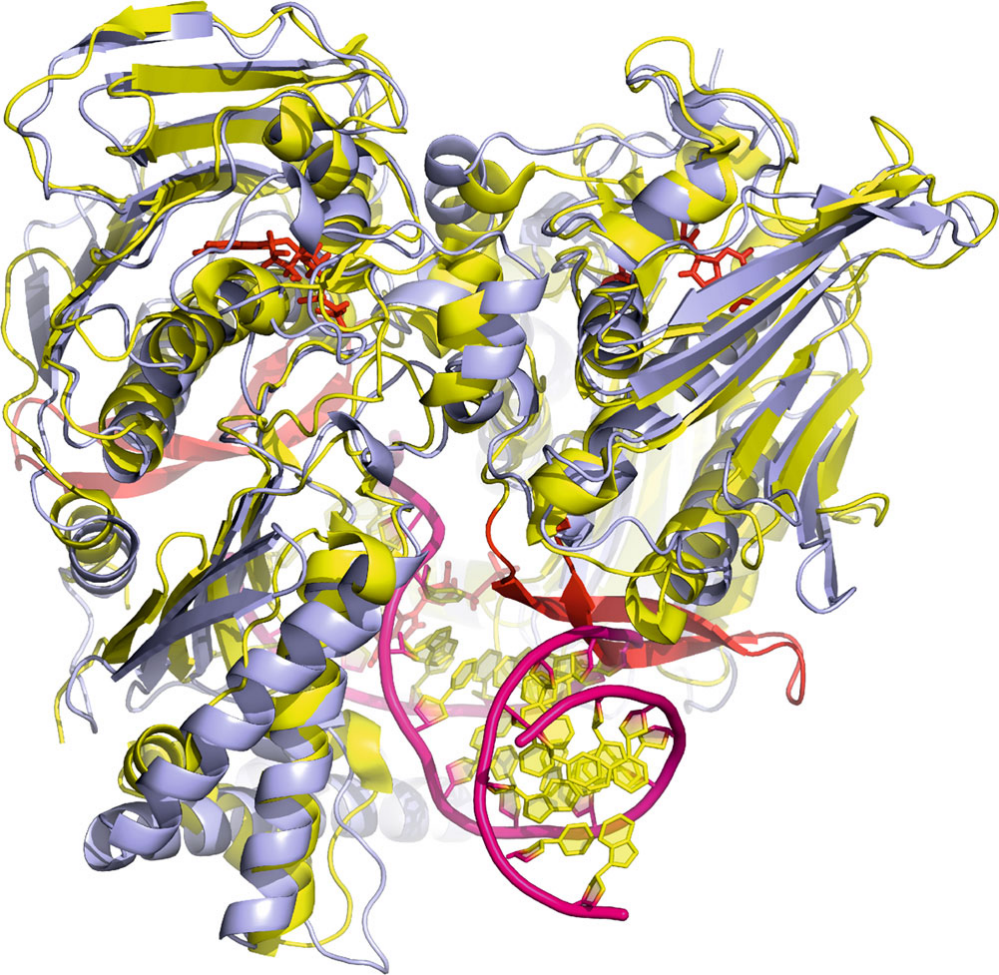
Overlap between the dimerized yeast topoisomerase II ATPase domain (Protein Data Bank 4GFH])^31^ (shown in yellow) and *S. pneumoniae* topoisomerase IV ATPase domain (through-hole DNA bound complex; shown in blue). AMP-PNP molecules are shown in red, and DNA is shown in pink/yellow for backbone/bases, respectively. The additional eukaryotic topo II-specific loop is shown in red. The structure indicates a potential clash between the bound DNA and the 22-amino acid long insertion loop in the eukaryotic topoisomerase II structure (Supplementary Fig. 8)

Our studies on the ParE44-DNA complexes identify both overlap and differences with other topoisomerase ATPase domains and cognate systems. There are some similarities in fold and some positively charged residues match in the sequence alignment with the RNA-bound protein of *Thermatoga maritima* RNAse P itself (Supplementary Fig. 7)^44^. However, RNAse P is much richer in Arg and Lys residues, makes many more contacts with the RNA and uses different residues compared with the binding of ParE to DNA. It appears that the RNAse P-like domain of ParE and the protein subunit of the ribonucleotide complex of RNAse P interact very differently with their nucleic acid partner. Sequence alignment of *S. pneumoniae* ParE44 with the ATPase domains of other type II topoisomerases reveals conservation and divergence in the residues interacting with DNA viz strong overall conservation of ParE R400; conservation among bacterial gyrases and topo IVs of K291, R321 and K346 but not in eukaryotic topoisomerases; and highly divergent residues corresponding to S268, D269, R353, R396 (Supplementary Fig. 8) pointing to potential differences in T-segment binding by eukaryotic type II enzymes. Furthermore, eukaryotic type II topoisomerases have a 22-residue insertion loop in the GHKL ATPase domain absent in prokaryotes (Supplementary Fig. 8) that drops down near the ATPase hole and could potentially clash with T-segment binding or transport (Fig. 8). Accordingly, eukaryotic topo II may kink the T-segment DNA more severely to allow its capture, require a conformational change of the loop or engage the T segment differently perhaps using the loop for DNA sequence sensing. Different to topo IV, we note that early studies on *E. coli* DNA gyrase showed that ATP hydrolysis was largely dependent on DNA length requiring a ≥70 bp DNA substrate but did not exclude preferred DNA sequences^33^. Our preliminary studies show that the 14-mer duplex effective with topo IV did not activate the gyrase ATPase, suggesting enzyme differences for gyrase perhaps linked to its requirement for DNA wrapping and intramolecular strand passage^13,33^. This information should aid the development of new antibacterial and anticancer agents targeting prokaryotic or eukaryotic topoisomerases.

In this paper, we reveal the first structure of a prospective T-segment DNA bound in the cavity formed by the closed ATPase clamp of a Top2A complex—a crucial intermediate in the Topo2A reaction pathway—as well as the first structure of a side-bound DNA-ParE complex that may cast light on how Top2As initially recognize the T segment. We show that the cavity-bound DNA is kinked and binds asymmetrically providing a structural rationale for the stepwise hydrolysis of the two ATP molecules. Comprehensive mutagenesis of cavity residues reveals strong effects on enzymatic DNA strand passage and ATP hydrolysis demonstrating biological relevance to the reaction pathway. Moreover, we show that short DNA duplexes activate the Top2A ATPase in a manner dependent on DNA conformation. Overall, we provide structural and biochemical insights that resolve a number of outstanding conundrums of Top2A mechanism that will stimulate new lines of experimentation and open new avenues for the discovery of novel therapeutic agents.

## Methods

### Plasmids and DNA substrates

Plasmid pET-29a was used as a vector for overexpression of wild-type and mutant ParE proteins in *E. coli* host BL21(λDE3)pLysS. Supercoiled pBR322 was obtained from New England Biolabs. Kinetoplast DNA (kDNA) was purchased from TOPOGEN, Inc. (Columbus, Ohio). All oligonucleotides including the 14-mer palindromic DNA sequence 5′‑GCATATATATATGC-3′ and 5′-GCGCGC-3′ were synthesized by solid-phase phosphoramidite chemistry and doubly HPLC purified by Metabion, Munich. DNA was dissolved in 20 mM Tris-HCl, pH 7.5, 200 mM NaCl, 1 mM 2-mercaptoethanol and 0.05% sodium azide at the final concentration of 1 mM. The oligomers were annealed or self-annealed by heating to 95 °C and slowly cooled over 2 nights in a Dewar flask kept in the cold room at 4 °C.

### Chemicals and reagents

Ciprofloxacin-HCl was provided by Bayer UK (Newbury, UK). β-Nicotinamide adenine dinucleotide (reduced form, dipotassium salt), pyruvate kinase, lactate dehydrogenase, phospho(enol)pyruvic acid monosodium salt hydrate and AMP-PNP were from Sigma Aldrich.

### Purification of *S. pneumoniae* ParE44

The duplex DNA encoding the ParE ATPase domain (UniProt Q59961, residues 1–402, plus 6xHis C-terminal tag) was synthesized by GENEWIZ and cloned into pET28b vector between *Nco*I and *Xho*I sites. *E. coli* BL21 λDE3 competent cells (Novagen) were transformed according to the manufacturer’s protocol. The cell culture was grown in LB broth at 30 °C for overexpression with constant agitation and induced with 1 mM isopropyl β-d-1-thiogalactopyranoside (IPTG; final concentration) at OD600 of 0.6. Kanamycin (50 µg ml^−1^) was included throughout the growth and expression period to prevent loss of the plasmid. Bacteria were grown for 8 h and then harvested by centrifugation. The cell pellet was subjected to one freeze–thaw cycle at −20 °C. After thawing, cells were disrupted by sonication, resuspended in lysis buffer (50 mM Tris-HCl, pH 7.5, 300 mM NaCl, 5 mM 2-mercaptoethanol, 50 mM imidazole, 0.05% sodium azide) and incubated on ice for 30 min. Lysozyme and ROCHE EDTA-free protease inhibitor tablets were added to the lysis buffer to give the recommended inhibitor concentrations. All the following purification steps were performed on ice or at 4 °C. Lysate was centrifuged in a Beckman Coulter JA-20 rotor at 18,000 rpm for 1 h. The pellet was discarded and the supernatant was loaded on to a freshly packed Ni-NTA column. The column was washed first with the lysis buffer (minus lysozyme) and then with the wash buffer (50 mM Tris-HCl, pH 7.5, 300 mM NaCl, 5 mM 2-mercaptoethanol, 80 mM imidazole, 0.05% sodium azide) until no protein could be detected in the flow-through (monitored using Bio-Rad solution). Elution was performed using the elution buffer (50 mM Tris-HCl, pH 7.5, 300 mM NaCl, 5 mM 2-mercaptoethanol, 200 mM imidazole, 0.05% sodium azide). The eluted protein was dialyzed overnight against 20 mM Tris-HCl, pH 7.5, 200 mM NaCl, 1 mM 2-mercaptoethanol and 0.05% sodium azide. Protein was measured using a NanoDrop spectrophotometer and the protein was concentrated up to 10 mg ml^−1^ using PEG 35,000 and Millipore dialysis tubing (10 kDa cut-off). The protein was further dialyzed overnight against 20 mM Tris-HCl, pH 7.5, 100 mM NaCl, 1 mM 2-mercaptoethanol and 0.05% sodium azide.

### Crystallization

The ParE44 protein was mixed with DNA in a 1:2.2 molar ratio and supplemented with 10 mM MgCl_2_. AMP-PNP was added to a final concentration of 2 mM and the mixture was allowed to incubate overnight. The crystallization mixture was then centrifuged at 10,000 rpm in a bench microfuge. Protein–DNA complex was crystallized by the sitting drop method using MRC-2 Wilden crystallization plates and a Mosquito^®^ robot from TTP Labtech (www.ttplabtech.com) to set the drops. Drops were formed by mixing 600 nl of the protein mixture and 400 nl of precipitant solution N31 (though-hole DNA bound complex) or N47 (side-bound DNA complex) from the Natrix screen (50 mM HEPES, pH 7.0, 5 mM MgCl_2_, 25% PEG MME 550 and 50 mM Tris-Cl, pH 8.5, 0.1 M KCl, 10 mM MgCl_2_, 30% PEG 400, respectively) from Hampton Research. Large crystals grew over a week. Crystals were harvested, transferred to the cryoprotectant (mother liquor +20–25% glycerol), monitored and adjusted using quick diffraction tests on an in-house X-ray source and then immediately mounted on Hampton pins and frozen in liquid nitrogen.

### Data collection and processing

Data collection was performed at the Diamond Synchrotron (Oxfordshire, UK), beamlines I02 (side‑bound DNA complex) and I04 (through-hole DNA bound complex). A total of 900 images were collected with 0.2° oscillation and exposure of 0.2 s at 100% transmission. Detector–Pilatus 6MF, wavelength was set to 0.91915 Å and 0.97949 Å, respectively. The data were automatically processed by Xia2/SCALA^45,46^. The space groups were established to be *C*222_1_ and *C*2 with cell dimensions being: *a* = 134.44 Å, *b* = 136.46 Å, *c* = 136.27 Å and *a* = 124.95 Å, *b* = 72.74 Å, *c* = 222.96 Å, respectively (see Table 1).

### Structure solution and refinement

The starting model for the ParE ATPase domain was generated using the Phyre2^47^ online server and *E. coli* gyrase B structure^48^ (1EI1) as a prototype. The structure of the hole-bound 14-mer-ParE44 complex was solved by molecular replacement using Phaser^49^ in CCP4^50^.

For the side-bound DNA complex, one biological dimer was found in the asymmetric unit, while the through-hole DNA-bound complex revealed two. The protein model was refined using phenix.refine^51^ with several rounds of manual correction in WinCoot^52^. The high-resolution structure of the side-bound DNA complex was used as a starting protein model for the through-hole-bound DNA complex with the side-bound DNA removed. For the through-hole DNA-bound complex, DNA density was clearly observed for one of the dimers, but not for the other. For the side-bound DNA complex, DNA molecules were clearly seen on both sides of the dimer. Our crystal lattice analysis indicated that the two dimers in the AU of the through-hole protein–DNA complex are located in different crystallographic environments, i.e., crystal lattice contacts would allow for one dimer to have both orientations of the through-hole-bound DNA (due to the twofold symmetry of the protein dimer), while in the case of the other dimer only one orientation of DNA could be accommodated without a clash with crystallographic symmetry-related molecules. The former dimer yielded no interpretable DNA density possibly due to a mixed population of the two orientations present, while the second dimer had a clear DNA density with its ends extending into the open solvent cavities of the crystal and forming no noticeable contacts with the neighbouring molecules. AMP-PNP molecules and the DNA model were manually added/built in with WinCoot^52^ according to the 2*F*_obs_−*F*_calc_ and *F*_obs_−*F*_calc_ maps and the structures were further refined using phenix.refine^51^ and several rounds of manual correction in WinCoot. Despite the restraints implemented, the final model for the through-hole complex exhibited somewhat lesser geometric quality compared to the high-resolution side-bound DNA complex (see Table 1). This can be explained by the lower resolution and more mobile regions with poorer electron density in the molecule, particularly in the region of the transiently bound DNA and in the regions of the solvent-exposed exterior loops. Ramachandran statistics for the hole-bound complex showed 86% of residues falling into favoured regions, 11% into allowed and 3% into outlier regions respectively with the clash score of 28. For the side-bound complex the percentages are of 97, 2 and 0.5% respectively for favoured, allowed and outlier regions with the clash score of 4.

Model quality was assessed using WinCoot (including geometry analysis and Ramachandran plot) as well as the verification tools provided by the Protein Data Bank. Figures for the paper were generated using PyMOL^53^, ChemDraw^54^ and CorelDraw (www.coreldraw.com).

### Expression of wild-type and mutant ParE and ParC proteins

*Streptococcus pneumoniae* ParE and ParC subunits were expressed from plasmids pXP13 and pXP14 that had been transformed into *E. coli* strain BL21(λDE3)pLysS and the recombinant proteins were purified to >95% homogeneity using a modification of published protocols^55–57^. Cultures were grown initially at 30 °C for around 3 h until the OD_600_ reached 0.3–0.4, and then shifted to 16 °C. Cultures were induced with 0.4 mM IPTG for 17 h. Cells were harvested, processed and the soluble ParE and ParC proteins were purified by nickel chelate chromatography^55–57^.

To generate ParE proteins with defined mutations in the ATPase region, the full-length *parE* gene in expression plasmid pXP13 was used as a template for site-directed mutagenesis using the QuikChange protocol and oligonucleotide pairs (39-mer to 47-mer) containing designed mutations (see Supplementary Table 1). All procedures were carried out according to the manufacturer’s instructions. Plasmids were recovered and sequenced to confirm the appropriate mutation had been successfully introduced. The mutant *parE* plasmids were then transformed into *E. coli* BL21(λDE3)pLysS. Induction and purification of soluble mutant- and wild-type-ParE proteins to >95% homogeneity by nickel chelate chromatography were carried out in parallel under identical conditions as described above^55–57^.

### Titration of ParE decatenation/relaxation activities

For decatenation of kDNA, the standard reaction mixture (20 µl) contained 40 mM Tris-HCl (pH 7.5), 6 mM MgCl_2_, 10 mM dithiothreitol (DTT), 200 mM potassium glutamate, 1 mM ATP, 50 µg ml^−1^ bovine serum albumin (BSA) and 400 ng of kDNA as substrate. A fixed amount of ParC subunit (25 ng) was combined with various amounts of ParE proteins. Reaction mixtures were incubated at 37 °C for 1 h, the reactions were terminated by addition of dye mix, and then the products were analysed by electrophoresis in 1% agarose.

Relaxation assays were carried out by a similar protocol except supercoiled plasmid pBR322 (400 ng) was used as substrate. Reaction mixes were incubated at 37 °C for 1 h, and the DNA products were examined by electrophoresis in 1% agarose gels.

### DNA cleavage assays

Reactions were set up using supercoiled pBR322 DNA (400 ng) as substrate. Topo IV reconstituted by combining ParC (450 ng) and either wt or mutant ParE (1 µg) was incubated with DNA in the absence or presence of ciprofloxacin at increasing concentrations. The reaction buffer was the same as for decatenation except ATP was omitted. Samples were incubated at 37 °C for 1 h followed by addition and mixing of 2 µl of 10% SDS to release cleaved DNA, and following addition of proteinase K to 200 µg ml^−1^, incubation was continued at 42 °C for 1 h to remove DNA-bound proteins. Sample loading dye (5 µl) was added to each tube. Cleavage products were separated and visualized by electrophoresis in 1% agarose.

### ATPase assays

ATP hydrolysis by wt and mutant topo IV complexes (130 nM ParC and 100 nM ParE) was carried out using supercoiled (or linear) pBR322 or defined oligonucleotide duplexes (10 µg) in decatenation buffer containing 2 mM ATP at 37 °C (final volume of 500 µl) using a standard assay^34,35^.

### Data availability

Coordinates have been deposited with the Protein Data Bank under accession numbers 5J5P and 5J5Q.

## Electronic supplementary material

Supplementary Information
