# Supplementary material for: Trapping of the transport-segment DNA by the ATPase domains of a type II topoisomerase
Source: Nat Commun. 2018 Jul 3;9:2579. doi: 10.1038/s41467-018-05005-x (PMC6030046; doi:10.1038/s41467-018-05005-x)
Supplement: Supplementary file 1 — Supplementary Information [file 41467_2018_5005_MOESM1_ESM.pdf]

Supplementary Information:

'Trapping of the Transport-segment DNA by the ATPase domains of a type II topoisomerase'

Ivan Laponogov[1,2†], Xiao-Su Pan[2], Dennis A. Veselkov[1], Galyna B. Skamrova[1], Trishant R. Umrekar[1‡], L. Mark Fisher[2]* and Mark R. Sanderson[1]*.

[1]Randall Centre for Cell and Molecular Biophysics, 3rd Floor New Hunt's House, Faculty of Life Sciences and Medicine, King's College London, London SE1 1UL, UK and [2]Molecular and Clinical Sciences Research Institute, St. George's, University of London, Cranmer Terrace, London SW17 0RE, UK.* Corresponding authors.

†Present address: Department of Surgery and Cancer, Faculty of Medicine, Sir Alexander Fleming Building, Imperial College London, London, SW7 2AZ, UK.

‡Present address: The Institute of Structural and Molecular Biology, Department of Biological Sciences, Birkbeck College, University of London, Malet St., London WC1E 7HX, UK.

Supplementary Figures 1 to 8

Supplementary Tables 1 and 2

Supplementary References

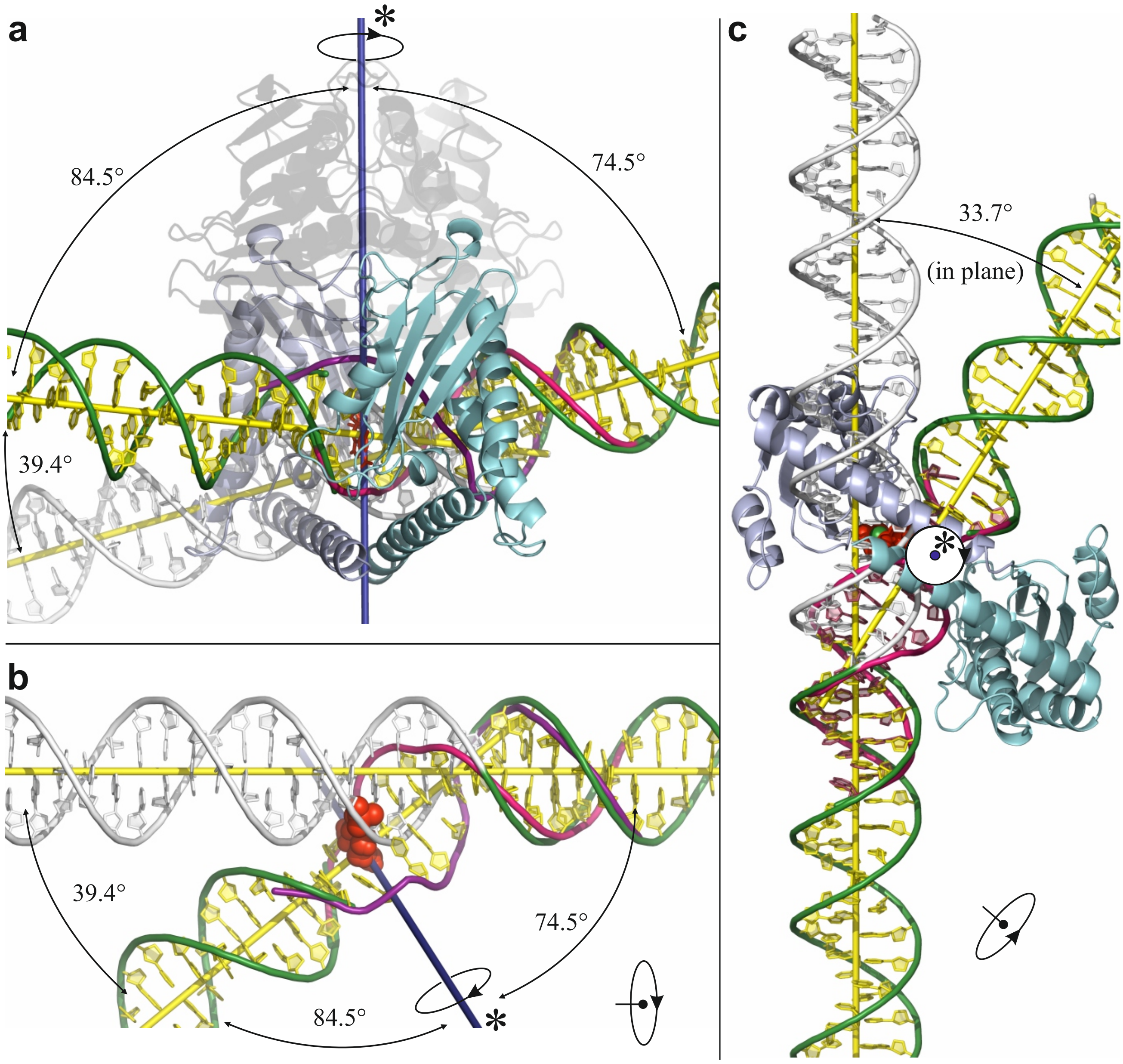

Supplementary Figure 1. Extrapolation of the T-segment DNA trajectory and its change due to the binding to the N-terminal ParE domains of topoisomerase IV**. (a)** View of the complex with the protein 2-fold rotational symmetry axis (shown in blue and indicated by an asterisk) oriented vertically, **(b)** View of the DNA projections and the T-segment bound DNA with the bending angle in the plane and **(c)** View from the bottom of the complex with the 2-fold rotational symmetry axis of the protein dimer (*) aligned perpendicular to the plane of the view. The total bending angle is 39.4° and the tilt from the 2-fold rotational symmetry axis of the protein dimer (*) is 74.5° and 84.5° accordingly. This corresponds to a total bending of the DNA of 21° relative to the protein symmetry axis (*) and 33.7° from he plane perpendicular to this axis. The DNA backbone of the projected DNA is in green, the straight B-form DNA-coinciding with the original trajectory of the incoming DNA- is in white, DNA spiral axes are in yellow, protein dimer 2-fold symmetry axis is in green and is marked with asterisk. The proposed AMP-PNP molecule is in red and is shown in van der Waals representation. The rest of the figure follows the same colouring scheme as Figure 2. The ideal B-form DNA was generated using WinCoot[1] and fitted in PyMOL[2] using backbone phosphates as anchoring points, 3 points per chain on the side of the DNA protruding further into the solution and 2 points per chain on the side of the DNA protruding less correspondingly. According to 3DNA analysis[3], the bound T-segment DNA remains in the B-form apart from the points of heavy bending where the canonical form cannot be determined.

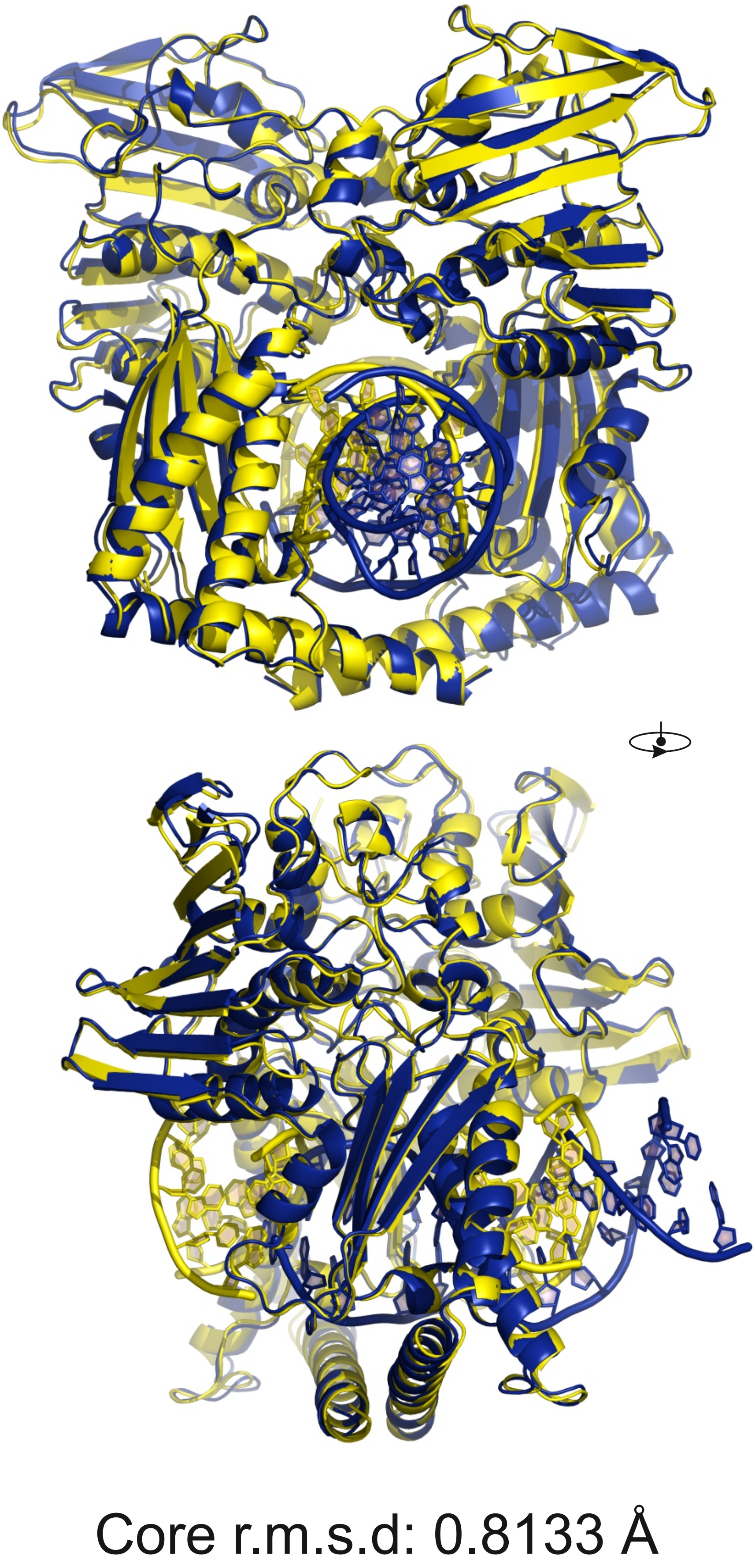

Supplementary Figure 2. Superposition of ParE complexes formed with hole-bound 14-mer and outside-bound 6-mer DNA duplexes (5J5Q and 5J5P, respectively). The superposition was performed in WinCoot [1] and the corresponding r.m.s.d. for the Cα carbons was 0.8133 Å. ParE-14mer and ParE-6mer backbones are shown in blue and yellow, respectively.

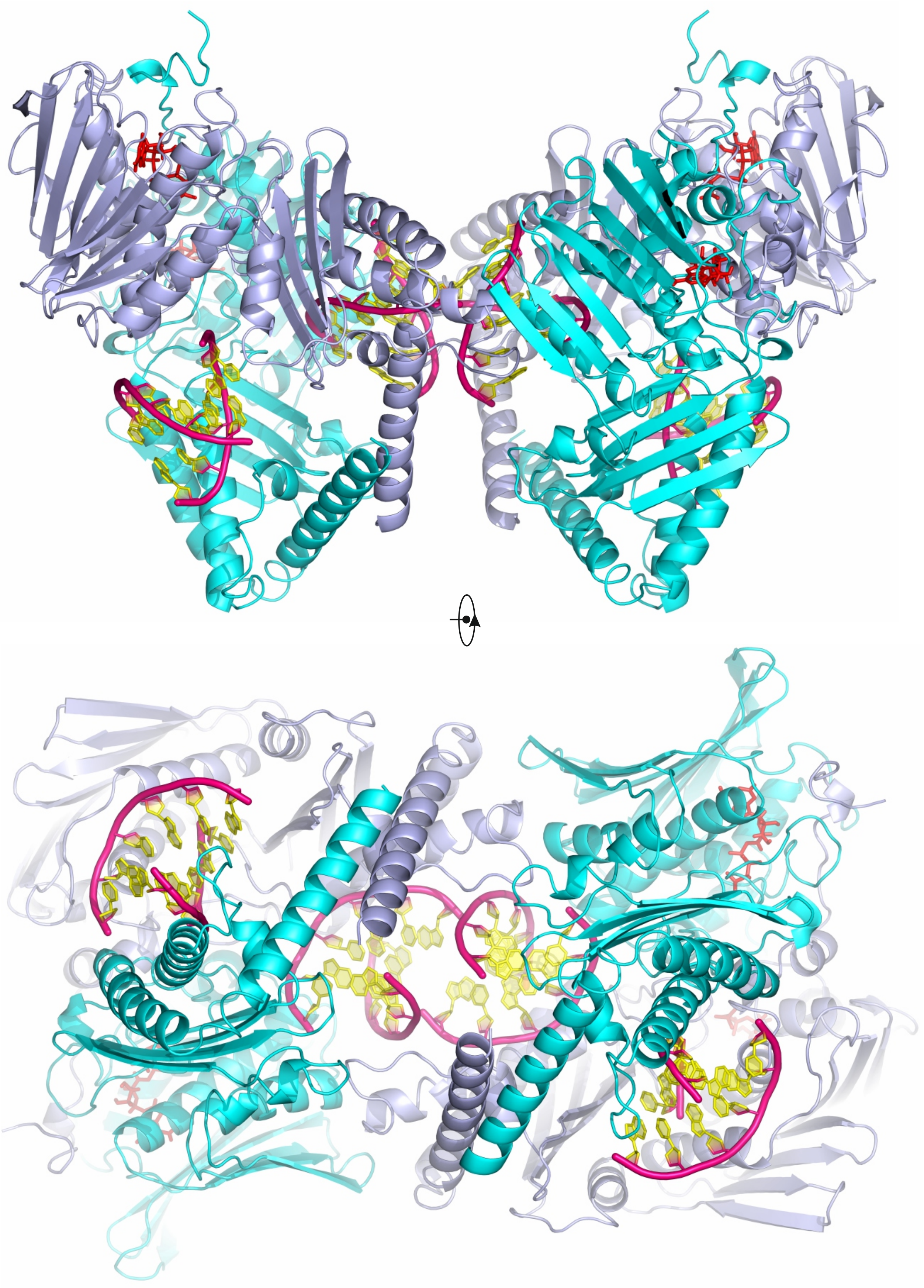

Supplementary Figure 3. Side-bound DNA-ParE44 complex structure exhibits a side-to-side contact between the bound DNA molecules from crystallographically-related protein dimers. Protein is in blue/cyan, DNA is in pink/yellow for backbone/bases, respectively. AMP-PNP molecules are in red.

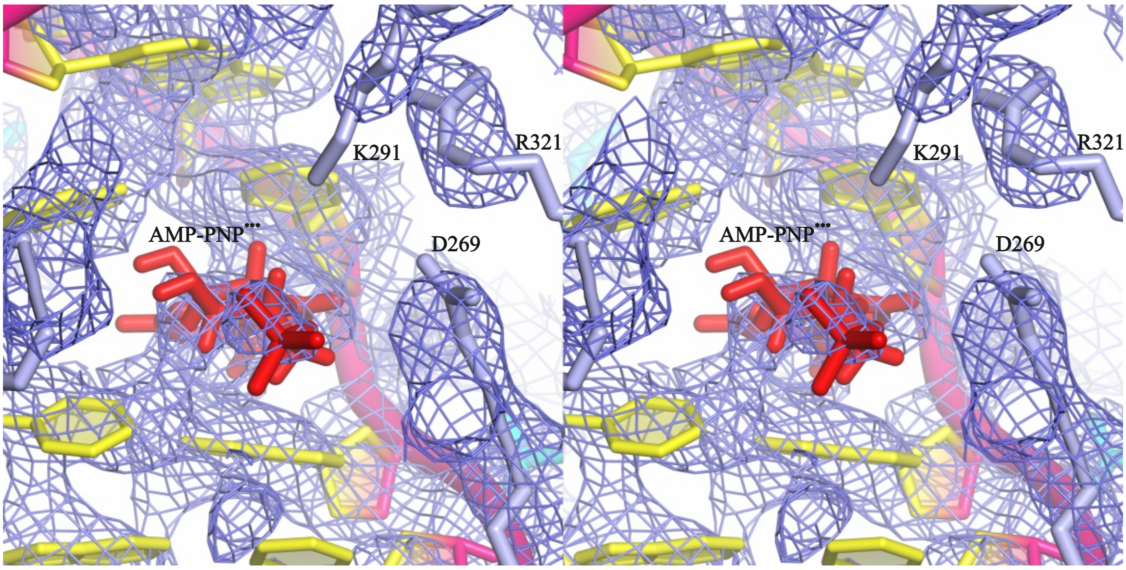

Supplementary Figure 4. Stereo image of the DNA intercalation site of the putative AMP-PNP molecule in the ParE44-14mer complex. AMP-PNP is in red, DNA backbone is in pink, DNA bases are in yellow, protein side chains involved in protein-DNA and protein-AMP-PNP contacts are in light blue/cyan. The composite omit map ($2F_{obs}$-F_{calc}) is contoured at the 1 sigma level.

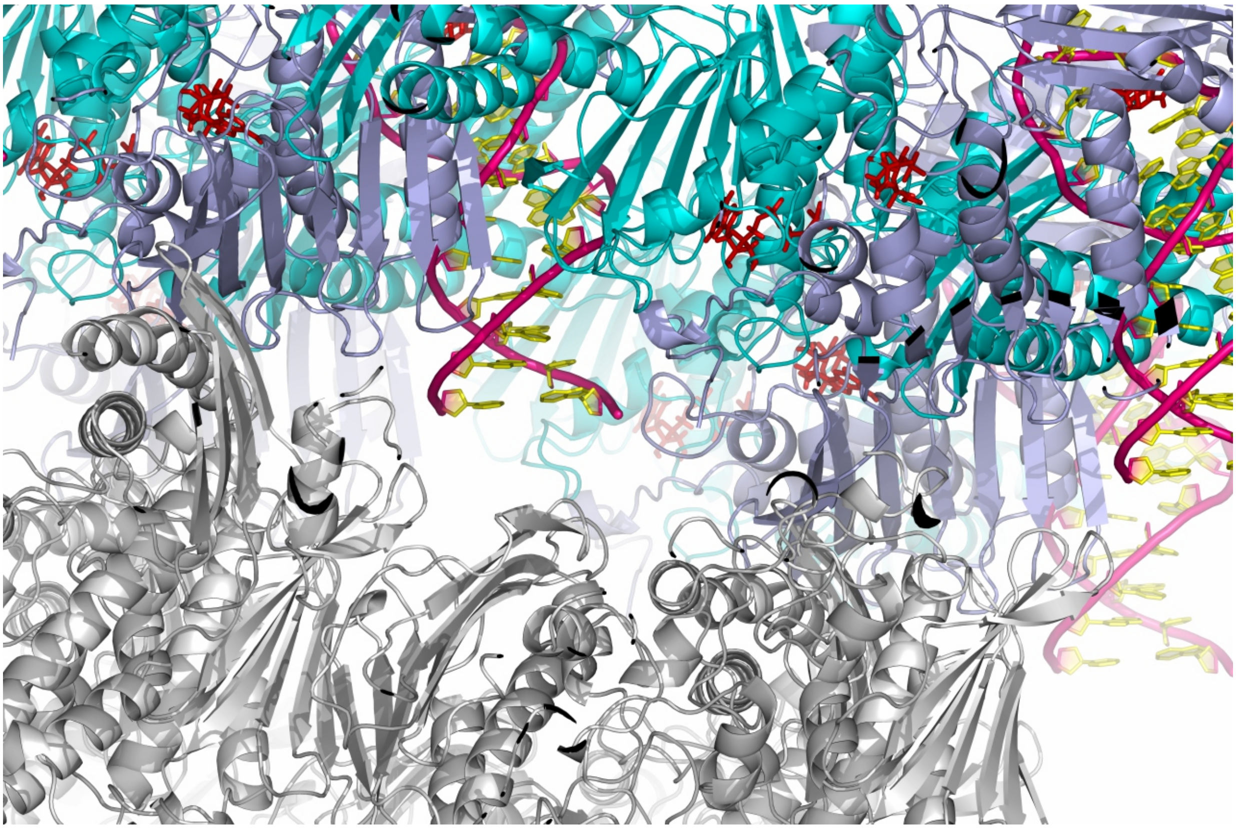

Supplementary Figure 5. Crystal lattice environment of the through-hole bound DNA-ParE44 complex. View through the crystal lattice onto the solvent pocket containing the longer DNA end protruding from the complex. The DNA only exhibits detectable contacts with the protein it is bound to and not to the crystallographically-related molecules. This is further reflected by the higher temperature factors for the longer protruding end of the DNA compared to the end which is bound to the protein (~260-350 $Å^2$ for the solvent exposed end vs ~190 $Å^2$ for the protein bound end). DNA-bound protein dimers are shown in blue/cyan. Protein dimers with no interpretable DNA density detected are in grey. The DNA is in pink/yellow for backbone/bases respectively. AMP-PNP molecules and the intercalated molecule X (putative AMP-PNP) are in red.

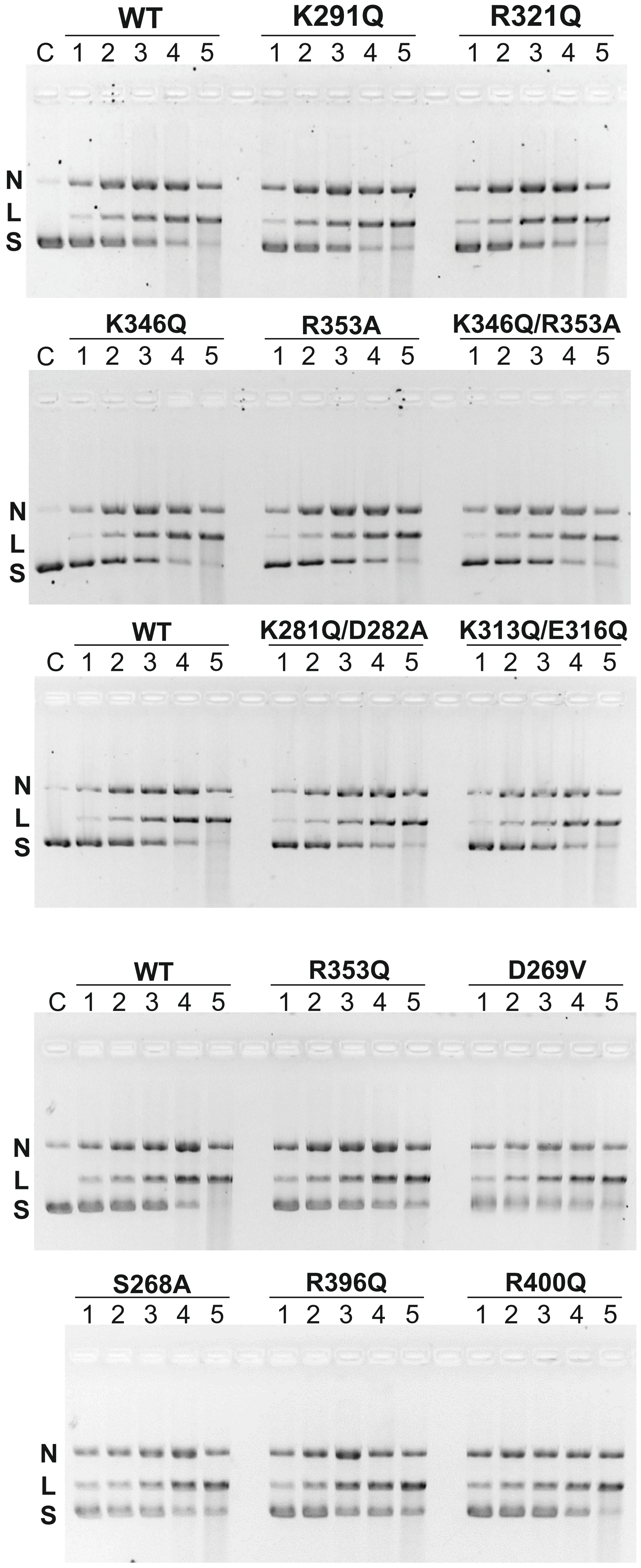

Supplementary Figure 6. Topoisomerase IV ParE cavity mutations do not affect DNA cleavage activity. For each panel of the figure, supercoiled plasmid pBR322 (400 ng) was incubated with *S. pneumoniae* ParC (450 ng) and either wild-type (WT) or mutant ParE (as indicated) (1 μg) at 37 °C for 1 hour in the absence (lane 1) or presence of ciprofloxacin at 1.2, 2.5, 5 and 10 μM (lanes 2-5, respectively). After denaturation with sodium dodecyl sulphate and proteinase K digestion, DNA products were separated and analysed by electrophoresis in 1% agarose gels. Lanes C, supercoiled DNA. N, L and S denote nicked, linear and supercoiled DNA.

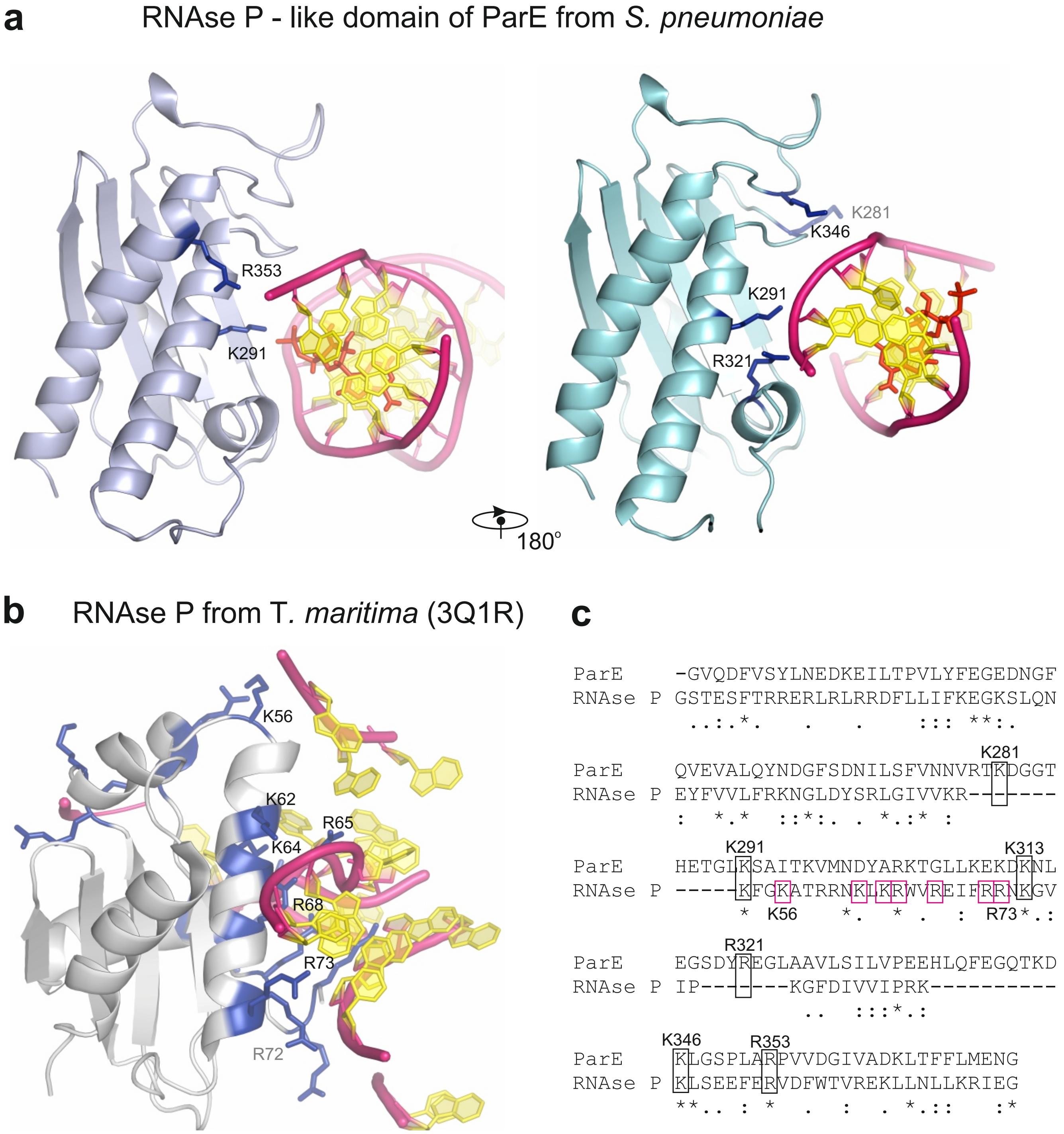

Supplementary Figure 7. Comparison between the RNAse P-like domain of ParE from *S. pneumoniae* bound to the 14-mer DNA and an RNA complex with RNAse P from *T. maritima* (PDB 3Q1R). (**a**) Both sides of the dimeric ParE are shown in the orientation equivalent to the one of RNAse P presented in (**b**). Residues with positively-charged side chains within 5 Å distance from the DNA/RNA backbone are shown in blue. (**c**) ClustalW-based sequence alignment[4] of the corresponding domains. Residues involved in DNA contacts in the ParE complex are indicated with the black boxes and amino acid labels at the top of the aligned sequences. Residues involved in RNA contacts within the equivalent region in RNAse P are highlighted with red boxes and amino acid labels at the bottom of the aligned sequences. DNA and RNA are shown in yellow/pink for bases and backbone, respectively. Proteins are shown in cartoon mode (ParE in light-blue and cyan, RNAse P protein is in light grey). The putative intercalated AMP-PNP is in red.

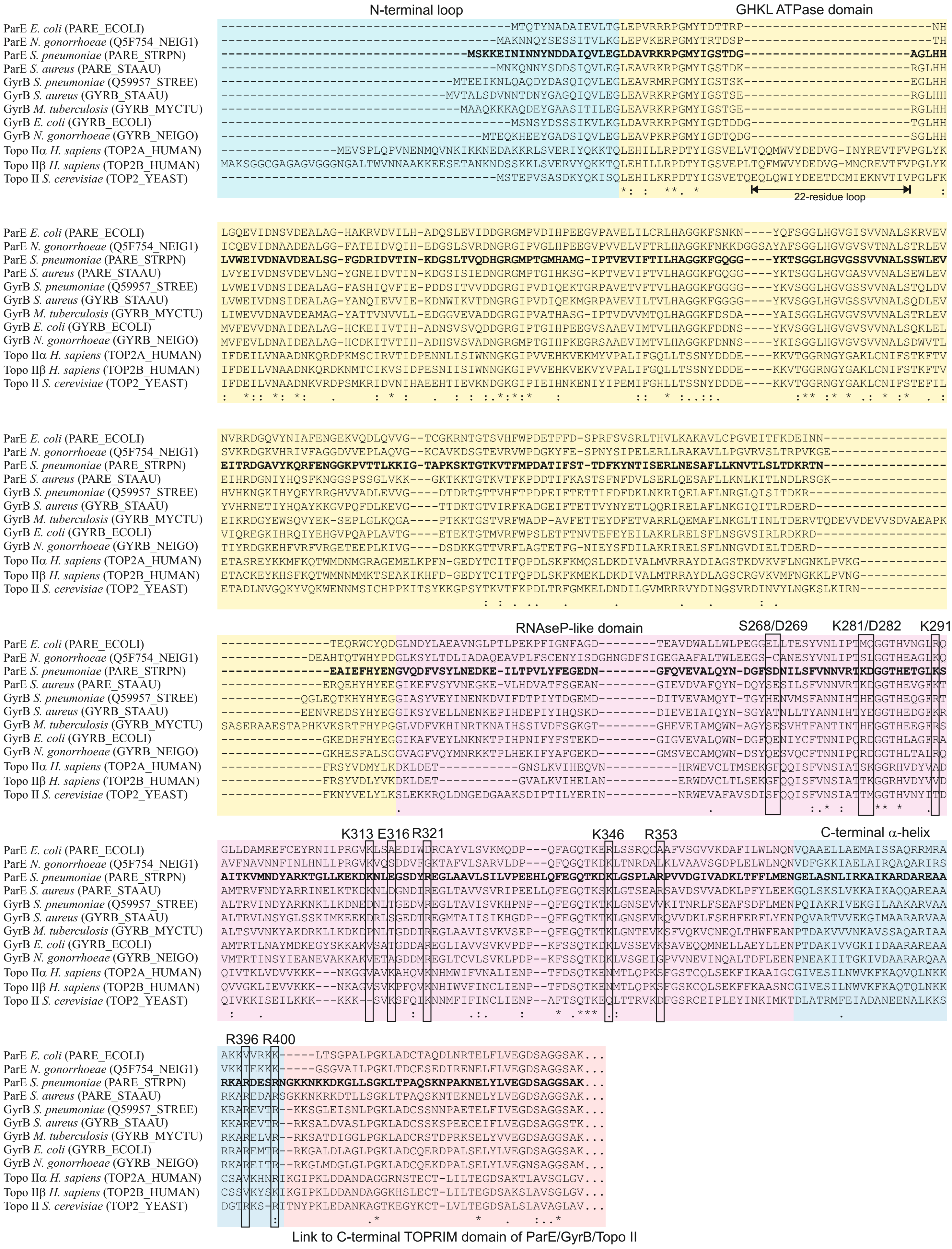

Supplementary Figure 8. Sequence comparison of the ATPase regions of ParE/GyrB or topo II from different organisms. Multi-sequence alignment was carried out using ClustalW[4]. The ParE sequence from *S. pneumoniae* is highlighted in bold. Twelve cavity residues in ParE targeted by mutagenesis in the present study and their equivalents in other topoisomerase ATPases are indicated by boxes.

Supplementary Table 1. Primer pairs used for *S. pneumoniae* ParE ATPase domain mutagenesis.

**Primer name**	**5’ – 3’ oligomer (39-47 mer)†**
SP-ParE 291K-Q(F)*	ACGCACGAGACAGGACTC**CAG**TCTGCCATTACCAAGGTC
SP-ParE 291K-Q(R)*	GACCTTGGTAATGGCAGA**CTG**GAGTCCTGTCTCGTGCGT
SP-ParE 321R-Q(F)	CTTGAAGGTTCAGACTAT**CAA**GAGGGACTAGCGGCCGTT
SP-ParE 321R-Q(R)	AACGGCCGCTAGTCCCTC**TTG**ATAGTCTGAACCTTCAAG
SP-ParE 346K-Q(F)	GACCAAGGAT**CAA**CTAGGAAGCCCCCTAGCTCGCCCAGTT
SP-ParE 346K-Q(R)	AACTGGGCGAGCTAGGGGGCTTCCTAG**TTG**ATCCTTGGTC
SP-ParE 353R-A(F)	GATAAACTAGGAAGCCCCCTAGCT**GCC**CCAGTTGTGGATG
SP-ParE 353R-A(R)	CATCCACAACTGG**GGC**AGCTAGGGGGCTTCCTAGTTTATC
SP-ParE 353R-Q(F)	CTAGGAAGCCCCCTAGCT**CAG**CCAGTTGTGGATGGAATA
SP-ParE 353R-Q(R)	TATTCCATCCACAACTGG**CTG**AGCTAGGGGGCTTCCTAG
SP-ParE 346K-Q, 353R-A(F)	GACCAAGGAT**CAA**CTAGGAAGCCCCCTAGCT**GCC**CCAGTTGTGGATG
SP-ParE 346K-Q, 353R-A(R)	CATCCACAACTGG**GGC**AGCTAGGGGGCTTCCTAG**TTG**TACCTTGGTC
SP-ParE 281K-Q, 282D-A(F)	GTCAATAACGTTCGCACC**CAGGCC**GGTGGAACGCACGAG
SP-ParE 281K-Q, 282D-A(R)	CTCGTGCGTTCCACC**GGCCTG**GGTGCGAACGTTATTGAC
SP-ParE 313K-Q, 316E-Q(F)	CAAGGAAAAAGAT**CAA**AACCTT**CAA**GGTTCAGACTATCG
SP-ParE 313K-Q, 316E-Q(R)	CGATAGTCTGAACC**TTG**AAGGTT**TTG**ATCTTTTTCCTTG
SP-ParE 269D-V(F)	TACAATGACGGATTCTCA**GTT**AACATTCTATCCTTTGTC
SP-ParE 269D-V(R)	GACAAAGGATAGAATGTT**AAC**TGAGAATCCGTCATTGTA
SP-ParE 268S-A(F)	CAGTACAATGACGGATTC**GCA**GATAACATTCTATCCTTT
SP-ParE 268S-A(R)	AAAGGATAGAATGTTATC**TGC**GAATCCGTCATTGTACTG
SP-ParE 396R-Q(F)	GAAGCAGCACGTAAGGCG**CAG**GATGAGAGCCGAAATGGG
SP-ParE 396R-Q(R)	CCCATTTCGGCTCTCATC**CTG**CGCCTTACGTGCTGCTTC
SP-ParE 400R-Q(F)	GAAGCAGCACGTAAGGCGCGTGATGAGAGC**CAA**AATGGG
SP-ParE 400R-Q(R)	CCCATT**TTG**GCTCTCATCACGCGCCTTACGTGCTGCTTC

*(F) and (R) denote forward and reverse primers.

†Altered codons are indicated in the primer name and are shown underlined and in bold.

Supplementary Table 2. Basal ATPase activity of wild-type and mutant *S. pneumoniae* ParE proteins is novobiocin-sensitive.

ParE	Inhibition of ParE ATPase by novobiocin (µM):	
	50% inhibition	Full inhibition
Wild-type	2.5-5	10
K291Q	10-20	>80
R321Q	2.5-5	10
K346Q	5-10	80
R353A	2.5-5	10
R353Q	2.5-5	20
D269V	5	>80
S268A	2.5-5	20
R396Q	5-10	80
R400Q	5-10	80

ATP hydrolysis was measured at 37°C in a coupled assay by the method of Tingey and Maxwell[5] using 2 mM ATP and 100 nM ParE protein in topo IV decatenation buffer (final volume 0.5 ml).

Supplementary References:

1. Emsley, P., Lohkamp, B., Scott, W. G. & Cowtan, K. Features and development of Coot. *Acta Crystallogr. D. Biol. Crystallogr.* **66**, 486-501 (2010).

2. DeLano, W. L. The PyMOL Molecular Graphics System. DeLano Scientific LLC, Palo Alto, CA, USA (2008).

3. Zheng, G., Lu, X.J. & Olson, W.K. Web 3DNA- a web server for the analysis, reconstruction and visualization of three-dimensional nucleic-acid structures. *Nucleic Acids Res.* **37**, W240-W246 (2009).

4. Larkin, M.A et al. Clustal W and Clustal X version 2.0. *Bioinformatics* **23**, 2947-2948 (2007).

5. Tingey, A.P. & Maxwell, A. Probing the role of the ATP-operated clamp in the strand passage reaction of DNA gyrase. *Nucleic Acids Res.* **24**, 4868-4873 (1996).
